# Structure and Haem-Distal Site Plasticity in *Methanosarcina acetivorans* Protoglobin

**DOI:** 10.1371/journal.pone.0066144

**Published:** 2013-06-12

**Authors:** Alessandra Pesce, Lesley Tilleman, Joke Donné, Elisa Aste, Paolo Ascenzi, Chiara Ciaccio, Massimo Coletta, Luc Moens, Cristiano Viappiani, Sylvia Dewilde, Martino Bolognesi, Marco Nardini

**Affiliations:** 1 Department of Physics, University of Genova, Genova, Italy; 2 Department of Biomedical Sciences, University of Antwerp, Antwerp, Belgium; 3 Interdepartmental Laboratory for Electron Microscopy, University Roma Tre, Roma, Italy; 4 Institute of Protein Biochemistry, National Research Council (CNR), Napoli, Italy; 5 Department of Clinical Sciences and Translational Medicine, University of Roma “Tor Vergata”, Roma, Italy; 6 Interuniversity Consortium for the Research on Chemistry of Metals in Biological Systems, Bari, Italy; 7 Department of Physics and Earth Sciences, University of Parma, Parma, Italy; 8 Department of Bioscience, University of Milano, Milano, Italy; 9 National Research Council-Biophysical Institute (CNR-IBF) and Interdisciplinary Centre for Nanostructured Materials and Interfaces (CIMaINa), University of Milano, Milano, Italy; Jacobs University Bremen, Germany

## Abstract

Protoglobin from *Methanosarcina acetivorans* C2A (*Ma*Pgb), a strictly anaerobic methanogenic Archaea, is a dimeric haem-protein whose biological role is still unknown. As other globins, protoglobin can bind O_2_, CO and NO reversibly *in vitro*, but it displays specific functional and structural properties within members of the hemoglobin superfamily. CO binding to and dissociation from the haem occurs through biphasic kinetics, which arise from binding to (and dissociation from) two distinct tertiary states in a ligation-dependent equilibrium. From the structural viewpoint, protoglobin-specific loops and a *N*-terminal extension of 20 residues completely bury the haem within the protein matrix. Thus, access of small ligand molecules to the haem is granted by two apolar tunnels, not common to other globins, which reach the haem distal site from locations at the B/G and B/E helix interfaces. Here, the roles played by residues Trp(60)B9, Tyr(61)B10 and Phe(93)E11 in ligand recognition and stabilization are analyzed, through crystallographic investigations on the ferric protein and on selected mutants. Specifically, protein structures are reported for protoglobin complexes with cyanide, with azide (also in the presence of Xenon), and with more bulky ligands, such as imidazole and nicotinamide. Values of the rate constant for cyanide dissociation from ferric *Ma*Pgb-cyanide complexes have been correlated to hydrogen bonds provided by Trp(60)B9 and Tyr(61)B10 that stabilize the haem-Fe(III)-bound cyanide. We show that protoglobin can strikingly reshape, in a ligand-dependent way, the haem distal site, where Phe(93)E11 acts as ligand sensor and controls accessibility to the haem through the tunnel system by modifying the conformation of Trp(60)B9.

## Introduction

Protoglobin (Pgb) is a recently discovered haem-protein with distinct structural and functional features that make it unique within the globin superfamily. All globins probably evolved from a common flavo-haemoglobin-like single-domain ancestral protein, and they can be classified phylogenetically along three main lineages [1]. Two of the lineages contain the chimeric flavohaemoglobins and related 3-on-3 globins (such as myglobin (Mb) and haemoglobin (Hb)), and the 2/2 globins, respectively, whereas Pgb belongs to a third lineage together with the globin coupled sensors (GCS) and the single domain sensor globins. However, within this lineage, while GCSs are chimeric haem-proteins, tentatively classified either as aerotactic or gene regulating, which couple a globin-like sensor domain to a transmitter domain of variable structure and function [2], Pgbs are instead single-domain variants without the transmitter domain. Up to now, more than nine Pgbs have been identified in both *Archaea* and *Bacteria* [1], [3]–[5]. Only two Pgbs have been characterized, from the obligate aerobic hyperthermophile *Aeropyrum pernix* [4], and from the strictly anaerobic methanogen *Methanosarcina acetivorans* [4], [6]. It is interesting to note that, despite the strict anaerobic nature of *M. acetivorans*, its genome hosts genes, such as *Pgb*, that can be related to the O_2_ metabolism. *Methanosarcinae* are metabolically and physiologically the most versatile methanogens, being able to exploit acetate, methanol, CO_2_ and CO as carbon sources for methanogenesis. This pathway is surprisingly simple and has been proposed to be the first metabolic pathway used by primordial microbes [7], [8]. In this context, Pgb could play a yet undisclosed role in the CO metabolism of these ancient organisms.

The only Pgb crystal structure reported so far is from *M. acetivorans*, (bearing the Cys(101)E20→Ser mutation, produced for crystallization purposes, hereafter termed simply *Ma*Pgb*) [6]. *Ma*Pgb* is similar, both in terms of tertiary structure and quaternary dimeric assembly, to the globin domain of the haem-based O_2_ sensor responsible for aerotaxis in aerobic *Bacillus subtilis* [9], and of GCS from the strictly anaerobic δ-Proteobacteria *Geobacter sulfurreducens* [10]. The *Ma*Pgb* structure can be considered as an expanded version of the 3/3 helical sandwich typical of “classical” globins (*i.e.* Mb), with an additional N-terminal extension, built by a 20-residue loop, followed by the Z-helix, which precedes the globin-fold conserved A-helix. Residues belonging to the Z-helix contribute to formation of the Pgb homodimer, which is centered on the intermolecular four-helix bundle built by the G- and H-helices of the two subunits [6]. The 20-residue N-terminal loop, together with other extended loops connecting the C-helix with the E-helix and the F-helix with the G-helix (which all are longer than in classical globins and conserved in Pgbs), completely bury the haem within the protein matrix, such that the haem propionates are solvent inaccessible (Figure S1). This structural feature, which is particularly unusual within the globin family structures, wherefore approximately 30% of the haem surface is normally solvent accessible, plays important role in ligand binding [11]. Thus, in Pgb the access of diatomic ligands, such as O_2_, CO, and NO, to the haem is granted by two orthogonal apolar tunnels that reach the haem distal region from entry sites at the B/G and B/E helix interfaces (Figure S1). These tunnels have no structural homologs within the globin family and are lined with residues highly conserved in all known Pgb sequences, suggesting functional implications for ligand diffusion to/from the haem cavity, for multi-ligand storage and/or for (pseudo-)enzymatic actions [6].

Other unusual trends in *Ma*Pgb* are the low O_2_ dissociation rate and a large structural distortion of the haem moiety [6]. Although it is generally accepted that the ligand dissociation rate constant is mainly determined by the interactions that stabilize the haem-bound ligand [12], in ferrous oxygenated *Ma*Pgb* (*Ma*Pgb*(II)-O_2_), the haem-bound O_2_ was found not to be stabilized by any hydrogen bond [6]. Remarkably, the unusually slow O_2_ dissociation rate constant in *Ma*Pgb* has been correlated to the large deviations from planarity of its porphyrin system [13]. The main out-of-plane contribution to the *Ma*Pgb* haem distortion is ruffling (which leaves opposite carbon atoms equally displaced and alternatively above and below the mean porphyrin plane), while the *Ma*Pgb* haem in-plane distortion is mainly ascribed to a strong breathing mode, which involves the symmetric compression-expansion of the porphyrin ring (with expansion associated to destabilization of the O_2_ binding energy, whereas the opposite trend is found for compression). Therefore, the haem compression due to the restricted *Ma*Pgb* heam binding pocket (Figure S1A), leads to a sizable stabilization of ligand binding, overcoming the destabilization due to ruffling, thus resulting in stabilization of the haem-bound O_2_, as compared to the ideal planar reference haem model [13]. The O_2_ binding behaviour of *Ma*Pgb* suggests a scenario where evolutionary events could subtly regulate the O_2_ affinity by shaping the haem cavity to favour out-of-plane distortions in order to decrease ligand affinity, or to compress the porphyrin ring to promote the reverse effect [13].

To shed light on the haem ligand binding mechanisms in such a peculiar globin, we present here the results of a crystallographic and kinetic investigation on ferric *Ma*Pgb* (*Ma*Pgb*(III)) and on selected mutants. In particular, the crystallographic investigation focuses on *Ma*Pgb*(III) complexed with cyanide, azide (both also in the presence of Xenon), imidazole, and nicotinamide. In parallel, kinetics show that, unlike what has been reported for O_2_ [6], the rate of cyanide dissociation is mainly determined by hydrogen bonding interactions that stabilize the haem-Fe(III)-cyanide complexes. A key conclusion, emerging from all experimental evidences here reported, is that *Ma*Pgb* can reshape strikingly the haem distal site structure, thus modulating accessibility to the haem through the tunnel system, depending on its (un)liganded state.

## Materials and Methods

### Expression and purification of MaPgb* and mutants

The *Ma*Pgb mutant bearing the Cys(101)E20Ser mutation (hereafter termed simply *Ma*Pgb*) was produced for crystallization purposes [14]. Mutations were introduced in *Ma*Pgb using the QuickChange™ site-directed mutagenesis method (Stratagene, La Jolla, CA). *Ma*Pgb* as well as the Trp(60)B9Ala, Tyr(61)B10Ala, Phe(93)E11Leu, Leu(142)G4Ala, and Ile(149)G11Phe mutants were expressed in *Escherichia coli* BL21(DE_3_)pLysS cells (Invitrogen, La Jolla, CA), and collected as described previously [14]. Refolding from inclusion bodies and purification of recombinant proteins were performed as described previously [6]. Briefly, the cells were exposed to three freeze-thaw steps and sonicated until completely lysed. Inclusion bodies were washed twice with 50 mM Tris-HCl (pH 8.0), 5 mM EDTA and 2% sodium deoxycholate, washed once with pure water, and solubilized by incubation in 100 mM Tris-NaOH (pH 12.0) and 2 M urea. After an incubation period of 30 min at room temperature and centrifugation at 10,700 *g* for 20 min at 4°C, proteins were refolded by adding 1.5 M of haemin. Then, after incubation of 10 min at room temperature, the pH was adjusted to 8.5 with HCl. The solution was then diluted into 5 volumes of distilled water and finally dialyzed at 4°C against the gel filtration buffer (50 mM Tris-HCl pH 8.5, 150 mM NaCl and 0.5 mM EDTA). Final purification was performed by gel filtration using a Sephacryl S200 column (GE Healthcare Europe GmbH, Diegem, Belgium) equilibrated with the gel filtration buffer.

### Crystallization and structure determination

The cyanide derivative of *Ma*Pgb*(III) and of its mutants was crystallized by vapor diffusion techniques (protein concentrations ∼45 mg/ml) under conditions matching those for the ligand-free *Ma*Pgb*(III) [6]. In particular, crystals of *Ma*Pgb*(III)-cyanide were grown by equilibrating the protein solution against a precipitant solution containing 30% PEG 4000, 0.2 M Li_2_SO_4_, 0.1 M Na Hepes (pH 7.0–7.5), 0.02 M potassium ferricyanide, and 0.01 M KCN (at 4°C). Crystals belong to the monoclinic space groups *P*2_1_ (two *Ma*Pgb* molecules in the asymmetric unit) or *C*2 (one *Ma*Pgb* molecule in the asymmetric unit). The best crystals diffracted to 1.6 Å resolution using synchrotron radiation (ESRF, Grenoble, France). Crystals of the cyanide-bound ferric Trp(60)B9Ala, Tyr(61)B10Ala, and Leu(142)G4Ala mutants were obtained by equilibrating the protein solutions (containing 0.005 M KCN) against 20–25% PEG 4000, 10% isopropanol, 0.1 M Na Hepes (pH 7.0–7.5), 0.02 M potassium ferricyanide, and 0.01 M KCN (at 4°C). All crystals belong to the primitive monoclinic *P*2_1_ space group (two *Ma*Pgb* molecules in the asymmetric unit) and diffracted to high resolution (in the 1.5 Å–1.7 Å range) using synchrotron radiation (ESRF, Grenoble, France). Crystallization conditions similar to those described above for the *Ma*Pgb*(III) mutants produced also crystals belonging to the monoclinic *C*2 space group (three *Ma*Pgb* molecules in the asymmetric unit) for the Phe(93)E11Leu, and Ile(149)G11Phe mutants, which diffracted up to 2.0 Å and 1.5 Å resolution, respectively, using synchrotron radiation (ESRF, Grenoble, France).

The azide derivative of *Ma*Pgb*(III) was prepared by adding to the *Ma*Pgb*(III) solution (about 43 mg/ml concentration) 0.01 M potassium ferricyanide and 0.1 M Na azide (NaN_3_). After 1 h of incubation, the protein-azide complexes were equilibrated against precipitant solutions containing 20–30% w/v PEG 4000, 0.2 M Li_2_SO_4_ or 10% isopropanol, 0.1 M Na Hepes (pH 7.0–7.5), at 4°C. The best *Ma*Pgb*(III)-azide crystals were grown either at 30% w/v PEG 4000, 0.2 M Li_2_SO_4_, 0.1 M Na Hepes (pH 7.5), or at 20% w/v PEG 4000, 10% isopropanol, 0.1 M Na Hepes (pH 7.0). In the first case, the crystals belong to the monoclinic *C*2 space group (two *Ma*Pgb* molecules in the asymmetric unit) and diffracted up to 1.8 Å, using synchrotron radiation (ESRF, Grenoble, France). In the second case, the crystals belong to the monoclinic *P*2_1_ space group (two *Ma*Pgb* molecules in the asymmetric unit); one of these was used for Xenon binding experiments. To promote Xenon diffusion within the protein matrix, the *Ma*Pgb*(III)-azide crystal was exposed to 10 bar Xenon for 5 min in a high-pressure chamber (Xcell, Oxford Cryo-system, UK), and rapidly transferred to liquid nitrogen. X-ray diffraction data up to 2.3 Å resolution were collected using synchrotron radiation (ESRF, Grenoble, France). An identical Xenon-binding procedure was also applied to the *Ma*Pgb*(III)-cyanide crystals (C2 crystal form) to produce the *Ma*Pgb*(III)-cyanide-Xenon complex.

The imidazole- and nicotinamide-bound *Ma*Pgb*(III) complexes were prepared by adding to the *Ma*Pgb*(III) solution (about 20 mg/ml concentration) 0.01 M potassium ferricyanide and 0.04 M either imidazole or nicotinamide. After 1 h of incubation, the *Ma*Pgb*(III)-ligand complexes were equilibrated against a precipitant solution containing 0.25–0.5 M monobasic ammonium phosphate or against 15–25% w/v PEG 4000, 10% v/v 2-propanol, and 0.1 M Na Hepes (pH 7.0–7.5), at 4°C. The best *Ma*Pgb*(III)-imidazole crystals grew in 0.4 M monobasic ammonium phosphate, matching the precipitant solution condition successfully used for the crystallization of the *Ma*Pgb*(II)-O_2_ complex [6]. The crystals belong to the monoclinic *C*2 space group (two *Ma*Pgb* molecules in the asymmetric unit) and diffracted up to 1.38 Å resolution using synchrotron radiation (ESRF, Grenoble, France). The best *Ma*Pgb(III)-nicotinamide crystals grew in 18% w/v PEG 4000, 10% v/v isopropanol, and 0.1 M Na Hepes (pH 7.5). They belong to the monoclinic *P*2_1_ space group (two *Ma*Pgb* molecules in the asymmetric unit) and diffracted up to 1.9 Å resolution using synchrotron radiation (ESRF, Grenoble, France). Statistics for each data collection are reported in details in Table S1 and Table S2.

All X-ray diffraction data were integrated and reduced using MOSFLM and SCALA [15], [16]; structure determination was achieved by molecular replacement methods with the program PHASER [17], using the *Ma*Pgb*(II)-O_2_ structure as the search model (PDB accession code 2VEB) [6]. Crystallographic refinement was performed using the program REFMAC [18], the program COOT [19] having been used for model building/inspection. The relevant refinement statistics are reported in Table S1 and Table S2. The program Procheck [20] was used to assess the stereochemical quality of the protein structures.

Atomic coordinates and structure factors have been deposited with PDB accession codes 3ZJN (*Ma*Pgb*(III)-cyanide complex), 3ZJR (*Ma*Pgb*(III)-cyanide-Xenon complex), 3ZJO (*Ma*Pgb*(III)-azide complex), 3ZJS (*Ma*Pgb*(III)-azide-Xenon complex), 3ZJP (*Ma*Pgb*(III)-imidazole complex), 3ZJQ (*Ma*Pgb*(III)-nicotinamide complex), 3ZJH (Trp(60)B9Ala-cyanide complex), 3ZJI (Tyr(61)B10Ala-cyanide complex), 3ZJJ (Phe(93)E11Leu-cyanide complex), 3ZJL (Leu(142)G4Ala-cyanide complex), and 3ZJM (Ile(149)G11Phe-cyanide complex).

### Determination of cyanide dissociation kinetics from MaPgb*(III)-cyanide and its mutants by reductive nitrosylation

Nitric oxide (NO) (from Aldrich Chemical Co., Milwaukee, WI, USA) was purified by flowing through an NaOH column in order to remove acidic nitrogen oxides. The NO stock solution was prepared by keeping the 2.0% borate buffer solution (pH = 9.2) in a closed vessel under NO at *P* = 760.0 mm Hg, anaerobically (*T* = 20.0°C). The solubility of NO in the aqueous buffered solution is 2.05×10^−3^ M, at *P* = 760.0 mm Hg and *T* = 20.0°C [21]. All the other products (from Merck AG, Darmstadt, Germany, and Sigma-Aldrich, St. Louis, MO, USA) were of analytical grade and used without purification unless stated.

The cyanide adducts of *Ma*Pgb*(III), and of the Trp(60)B9Ala, Tyr(61)B10Ala, Phe(93)E11Leu, Leu(142)G4Ala, and Ile(149)G11Phe mutants were obtained by adding a 10-molar excess of a cyanide stock solution (1.0×10^−3^ M) to the protein solutions (ranging between 4.4×10^−6^ M and 5.2×10^−6^ M) [21].

Values of the first-order rate constant for cyanide dissociation (*k*_off_) from *Ma*Pgb*(III)-cyanide and the mutant-cyanide complexes were determined by mixing the ferric protein-cyanide solutions with the NO solution under anaerobic conditions, at pH 9.2 (2.0% borate buffer) and 20.0°C; no gaseous phase was present. The final concentration of *Ma*Pgb*(III) and mutants ranged between 2.2×10^−6^ M and 2.6×10^−6^ M. The final cyanide concentration was ∼2.0×10^−5^ M. The final NO concentration ranged between 1.0×10^−4^ M and 1.0×10^−3^ M.

Kinetics of cyanide dissociation from *Ma*Pgb*(III)-cyanide and the mutant-cyanide complexes were analyzed in the framework of the minimum reaction mechanism represented by Scheme 1 [21], [22]:
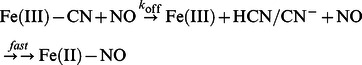



Of note: (*i*) cyanide dissociation from *Ma*Pgb*(III)-cyanide is the rate limiting step of the whole reductive nitrosylation process (present study); (*ii*) the *Ma*Pgb*(II)-NO complex is very stable, dissociating very slowly [22]; and (*iii*) cyanide binding to *Ma*Pgb*(II) is negligible at [cyanide] ∼2.0×10^−5^ M (unpublished data).

Depending of the observation wavelength, values of *k*_off_ were determined from data analysis according to Eqns. 1 and 2 [21], [22]:
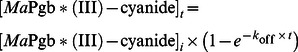
(1)

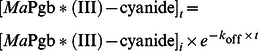
(2)where [*Ma*Pgb*(III)-cyanide]*_t_* is the cyanide-bound haem-protein(III) concentration at time *t*, and [*Ma*Pgb*(III)-cyanide]*_i_* is the initial cyanide-bound haem-protein(III) concentration (*i.e.*, at *t* = 0).

Kinetics was monitored spectrophotometrically between 380 nm and 460 nm. The results are reported as mean values of at least four experiments, plus or minus the corresponding standard deviation. All data were analyzed using the matlab program (The Math Works Inc., Natick, MA, USA).

## Results

### Cyanide binding mode to MaPgb*(III) and to the mutant MaPgbs*

High-resolution data (1.60 Å) on the *Ma*Pgb*(III)-cyanide crystals were collected at the ESRF synchrotron facility (ESRF, Grenoble, France), and the structure was refined to a final R-factor and R-free of 17.5% and 23.8%, respectively (Table S1). The tertiary structure of *Ma*Pgb*(III)-cyanide is nearly identical in its backbone to those of ligand-free *Ma*Pgb*(III) and *Ma*Pgb*(II)-O_2_ [6], displaying rms deviation values that range between 0.37 Å and 0.56 Å, calculated for 190 Cα atom pairs. Similarly, the quaternary assembly of the two independent *Ma*Pgb*(III)-cyanide molecules present in the crystal asymmetric unit (A and B chains) closely matches the dimer assembly of ligand-free *Ma*Pgb*(III) and of *Ma*Pgb*(II)-O_2_ [6].

**Figure 1:**
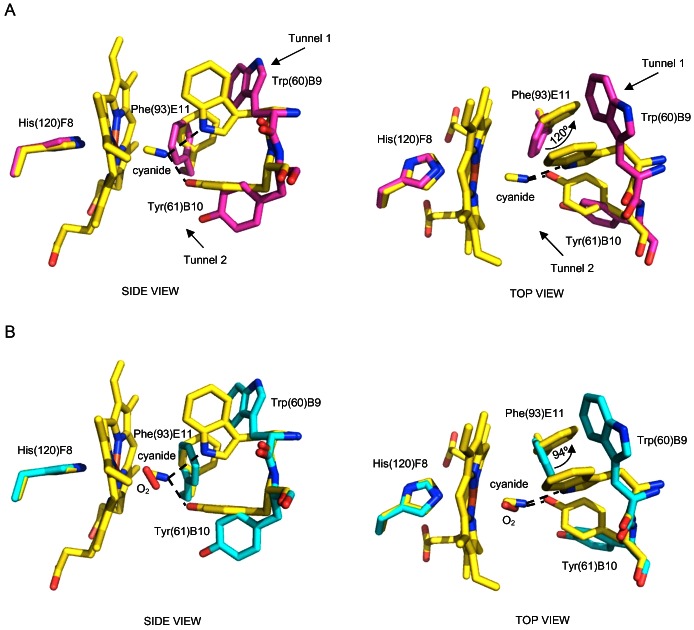
The haem distal site of *Ma*Pgb*(III)-cyanide. Residues lining the haem distal pocket are indicated and shown in stick representation (yellow). Superimposition of *Ma*Pgb*(III)-cyanide to (**A**) ligand-free *Ma*Pgb*(III) (magenta), and (**B**) *Ma*Pgb*(II)-O_2_ (cyan). The proximal His(120)F8 residue is also shown. Amino acid residues have been labeled using their three-letter codes, the sequence numbering (in parentheses), and the topological site they occupy within the globin fold. In panel (**A**) the haem distal cavity entrance sites of tunnel 1 and tunnel 2 are indicated by arrows. Both panels are shown from a side and a top view. Rotation of the Phe(93)E11 side chain upon ligand binding is indicated in each top view panel. H-bonds to the haem-Fe(III)-bound cyanide are indicated by dashed lines.

In the *Ma*Pgb*(III)-cyanide complex the cyanide molecule is bound to the sixth coordination site of the haem-Fe(III) atom, with a coordination bond of 2.16 Å and a Fe-C-N angle of 171° for chain A, and of 2.14 Å and 169° for chain B. Two H-bonds stabilize the haem-Fe(III)-bound cyanide molecule. On one hand, the cyanide N atom is linked to Tyr(61)B10 OH group (2.84 Å and 2.71 Å, for chain A and B, respectively), on the other, it is hydrogen bonded to Trp(60)B9 Nε2 atom (2.98 Å or 2.99 Å) (Figure 1A, left panel, and Figure S2A). When compared to the ligand-free *Ma*Pgb*(III) and the *Ma*Pgb*(II)-O_2_ structures some major differences appear evident. The presence of the haem-Fe(III)-bound cyanide molecule induces a rotation of the Phe(93)E11 side chain around the Cβ-Cγ bond of about 120° relative to the ligand-free *Ma*Pgb*(III) (Figure 1A, right panel).

**Figure 2:**
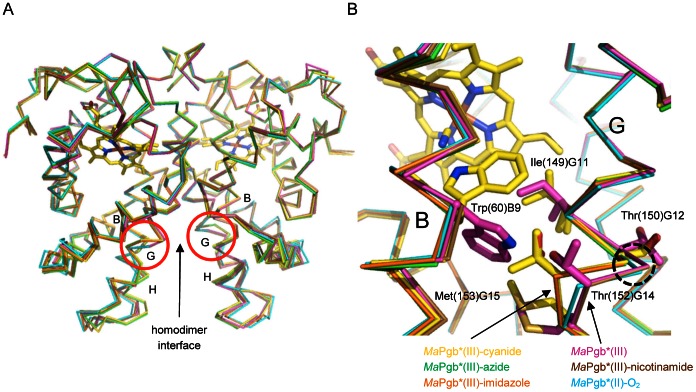
Superimposition of *Ma*Pgb*(III) structures in complex with different ligands. (**A**) Superimposition of homodimeric ligand-free *Ma*Pgb*(III) (magenta) onto *Ma*Pgb*(III)-cyanide (yellow), *Ma*Pgb*(III)-azide (green), *Ma*Pgb*(III)-imidazole (orange), *Ma*Pgb*(III)-nicotinamide (brown), and *Ma*Pgb*(II)-O_2_ (cyan). The subunit-subunit interface is indicated and relevant helices labeled. Red circles highlight the position of the 149–154 region in both subunits. (**B**) The 149–154 region in one *Ma*Pgb* subunit. For clarity only the side chains of Ile(149)G11, Thr(150)G12, Thr(152)G14, and Met(153)G15 from the ligand-free *Ma*Pgb*(III) (magenta) and *Ma*Pgb*(III)-cyanide (yellow) structures are compared, as representative of the two haem distal site open and closed conformations, respectively. The corresponding different orientations of the Trp(60)B9 side chain are also shown. The maximum Cα-backbone displacement at Ala(151)G13 is highlighted by a black dotted circle. For clarity, the side chain and label of Ala(151)G13 are omitted.

The rotation of the Phe(93)E11 side chain allows the rotation of the Trp(60)B9 side chain of ∼90° towards the center of the haem distal site. As a consequence, in the *Ma*Pgb*(III)-cyanide complex: (*i*) tunnel 1 is hindered by the Trp(60)B9 side chain, and (*ii*) the Trp(60)B9 Nε1 atom is at H-bond distance to the haem-Fe(III)-bound cyanide ligand (Figure 1A). In the *Ma*Pgb*(II)-O_2_ structure, where the haem-Fe(II)-bound ligand is not stabilized by any H-bond [6], the rotation of the Phe(93)E11 side chain is only ∼94° relative to ligand-free *Ma*Pgb*(III) (Figure 1B, right panel); this is not sufficient to allow the Trp(60)B9 side chain to enter the haem distal site and H-bond the ligand (Figure 1B). Interestingly, the 149–154 region, which faces Trp(60)B9 side chain on one side and the second subunit of the dimer on the other, is significantly divergent in its Cα-backbone relative to the ligand-free *Ma*Pgb*(III) and *Ma*Pgb*(II)-O_2_ structures (maximum displacement at Ala(151)G13 of 1.75 Å and 1.64 Å, respectively), and shows signs of structural heterogeneity, with Ile(149)G11 and Thr(152)G14 adopting two alternate conformations (Figure 2). Thus, ligand binding, and the consequent relocation of the Trp(60)B9 side chain within the haem distal cavity, appears to release structural constraints at the dimeric interface.

Cyanide binding causes also a shift of about 1 Å of the Tyr(61)B10 backbone toward the interior of the haem distal site, with the concomitant side chain rotation of ∼90° around the Cβ-Cγ bond. In this new orientation, the Tyr(61)B10 OH group provides a tight H-bond to the haem-Fe(III)-bound cyanide (Figure 1A). Notably, when H-bonded to the cyanide ligand, Tyr(61)B10 loses the H-bonds between its OH-group and the Arg(90)E8 amide N and the Leu(86)E4 carbonyl O atoms occurring in the ligand-free *Ma*Pgb*(III) and *Ma*Pgb*(II)-O_2_ structures [6].

**Figure 3:**
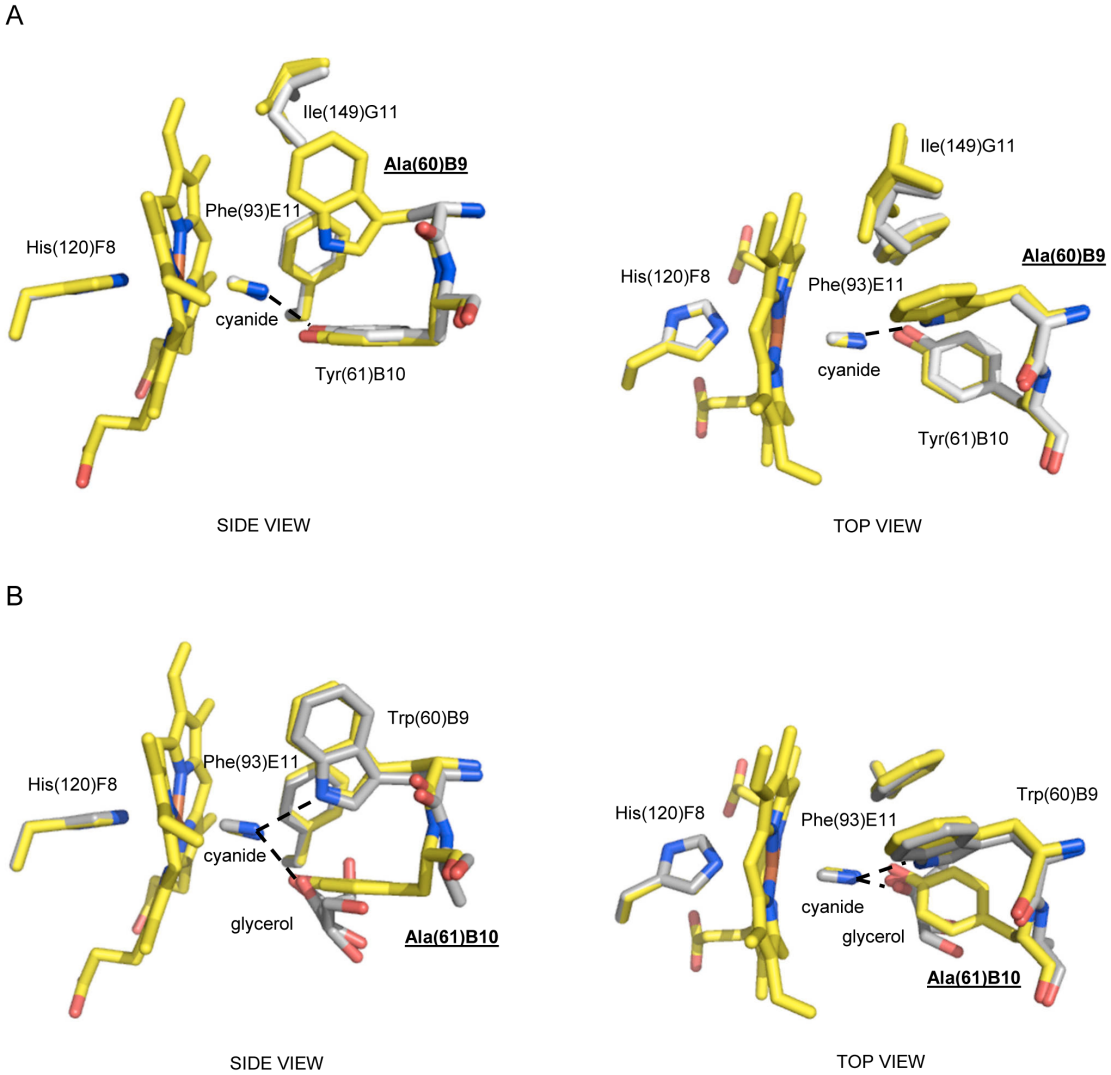
The haem distal site of *Ma*Pgb*(III)-cyanide Trp(60)B9Ala and Tyr(61)B10Ala mutants. Residues lining the haem distal pocket are indicated and shown in stick representation (grey). Superimposition of *Ma*Pgb*(III)-cyanide (yellow) onto (**A**) the *Ma*Pgb*(III)-cyanide Trp(60)B9Ala mutant, and (**B**) the *Ma*Pgb*(III)-cyanide Tyr(61)B10Ala mutant. The proximal His(120)F8 residue is also shown. H-bonds to the haem-Fe(III)-bound cyanide are indicated by dashed lines. The mutated residues are indicated in underlined bold characters. Both panels are shown from side and top views.

The sites of *Ma*Pgb*(III) mutations (Trp(60)B9Ala, Tyr(61)B10Ala, Phe(93)E11Leu, Leu(142)G4Ala, and Ile(149)G11Phe) are located in the surroundings of the haem, or along the two tunnels; the mutations were designed to evaluate the (structural) impact of the selected residues on the stabilization of the haem-Fe(III)-bound ligand and on the reshaping of the haem distal cavity/tunnel system. All mutant structures have been solved at high resolution, ranging from 1.5 Å to 2.0 Å (data statistics are reported in Table S2). Overall, the single mutations do not affect significantly the tertiary structure of the mutated proteins, which are always closely similar in their backbone to that of *Ma*Pgb*(III)-cyanide, displaying rms deviation values which range between 0.17 Å and 0.49 Å, calculated for 190 Cα atom pairs. Significant changes in the structure of the haem distal site and of the tunnels are however present in the case of the Trp(60)B9Ala and Tyr(61)B10Ala mutants. In the Trp(60)B9Ala mutant one cyanide stabilizing H-bond is absent, while the H-bond to Tyr(61)B10 OH group is maintained (2.82 Å for chain A, and 2.87 Å for chain B). As a result, the cyanide ligand is slightly tilted toward the entrance of tunnel 2, where Tyr(61)B10 is located, with a haem-Fe(III) coordination bond of 2.25 Å and a Fe-C-N angle of 151° for chain A, and 2.27 Å and 122° for chain B (Figure 3A). The Trp(60)B9Ala mutation sets tunnel 1 in a constantly open state. Only a minor rearrangement of the Ile(149)G11 side chain occurs within tunnel 1, which is however not sufficient to fill the empty volume left by the Trp→Ala mutation (Figure 3A, right panel). Interestingly, the location of the Phe(93)E11 side chain in the Trp(60)B9Ala mutant is superimposable to that of the Phe(93)E11 in the *Ma*Pgb*(III)-cyanide complex, thus indicating that the presence of the haem-Fe(III)-bound cyanide is enough to induce the Phe(93)E11 side chain rotation; it also suggests that the insertion of Trp(60)B9 side chain into the haem distal site is only a consequence of the cyanide binding to *Ma*Pgb*(III) and of the ligand-linked conformational change of Phe(93)E11 side chain.

The Tyr(61)B10Ala mutation has instead a dramatic impact on the accessibility of the haem distal site since it increases the average tunnel 2 diameter by more than 1.5 Å (6.0 Å is the shortest distance between Ala(61)B10 Cβ atom and the surrounding residues). As a result of such increased accessibility, the tunnel 2 entrance site (*i.e.* roughly the cavity left by the Tyr→Ala mutation) in the Tyr(61)B10Ala mutant hosts a glycerol molecule (in both chains A and B; the glycerol molecule bound to chain A is modeled in a double conformation) which is able to H-bond the haem-Fe(III)-bound cyanide (distance of ∼2.8–2.9 Å, depending on the protein chain) (Figure 3B). Thus, in the Tyr(61)B10Ala mutant, the haem-Fe(III)-bound cyanide molecule is stabilized by the interaction with the Trp(60)B9 Nε2 atom (2.92 Å for chain A, and 2.90 Å for chain B), and by the additional H-bond provided by the glycerol molecule which mimics the H-bond provided by the Tyr(61)B10 OH group in the *Ma*Pgb*(III)-cyanide structure (Figure 3B). As a result, both the orientations of the haem-Fe(III)-bound cyanide molecule and of the Phe(93)E11 side chain match those found in the *Ma*Pgb*(III)-cyanide structure.

**Figure 4:**
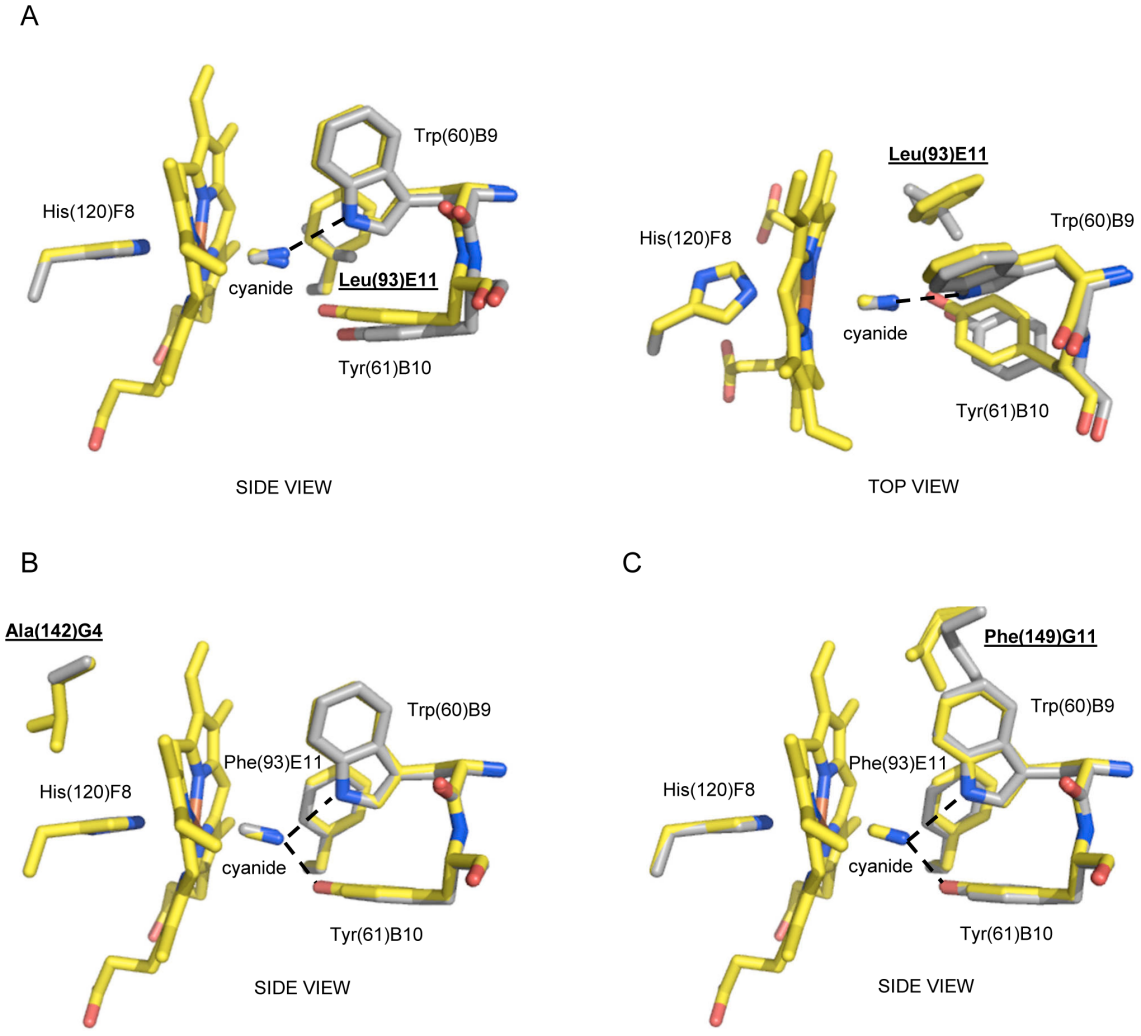
The haem distal site of *Ma*Pgb*(III)-cyanide Phe(93)E11Leu, Leu(142)G4Ala, and Ile(149)G11Phe mutants. Residues lining the haem distal pocket are indicated and shown in stick representation (grey). Superimposition of *Ma*Pgb*(III)-cyanide (yellow) onto (**A**) the *Ma*Pgb*(III)-cyanide Phe(93)E11Leu mutant (side and top views), (**B**) the *Ma*Pgb*(III)-cyanide Leu(142)G4Ala mutant (side view), and (**C**) the *Ma*Pgb*(III)-cyanide Ile(149)G11Phe mutant (side view). The proximal His(120)F8 residue is also shown. H-bonds to the haem-Fe(III)-bound cyanide are indicated by dashed lines. The mutated residues are indicated in underlined bold characters.

The Phe(93)E11Leu mutation does not prevent the insertion of the Trp(60)B9 side chain into the distal site and the stabilization of the haem-Fe(III)-bound cyanide (Figure 4A). Thus, the ligand sensing capability of the E11 residue, reflected by Trp(60)B9 conformational changes, does not require specifically a Phe residue. In the Phe(93)E11Leu mutant structure (three *Ma*Pgb* chains in the asymmetric unit), however, the haem-Fe(III)-bound cyanide molecule is stabilized only by the H-bond provided by the Nε2 atom of Trp(60)B9 (∼2.8 Å). Indeed, the orientation of the Tyr(61)B10 side chain matches that found in the *Ma*Pgb(III)-cyanide structure, but its side chain is slightly shifted away from the center of the haem distal cavity, its OH group being at ∼4 Å from the cyanide N atom. In this orientation, the Tyr(61)B10 OH group is not involved in any H-bond interaction, contrary to what is found in the ligand-free *Ma*Pgb*(III) and *Ma*Pgb*(II)-O_2_ structures, where the Tyr(61)B10 side chain, while not interacting with any ligand, is H-bonded to the Arg(90)E8 amide N and the Leu(86)E4 carbonyl O atoms [6].

The other two mutations considered, Leu(142)G4Ala, localized at the proximal site just on top of His(120)F8, and Ile(149)G11Phe, adjacent to Trp(60)B9 in tunnel 1, do not produce any significant deviation in the overall conformation of the distal site and in the cyanide binding mode relative to the *Ma*Pgb*(III)-cyanide structure (Figures 4B and C).

### Kinetics of cyanide dissociation from MaPgb*(III)-cyanide and its mutants by reductive nitrosylation

Mixing of cyanide-bound *Ma*Pgb*(III), as well as of Trp(60)B9Ala, Tyr(61)B10Ala, Phe(93)E11Leu, Leu(142)G4Ala, and Ile(149)G11Phe mutant solutions with NO solutions induced a shift of the absorption peak maxima from 420–422 nm (ε ranging between 110 mM^−1^ cm^−1^ and 120 mM^−1^ cm^−1^) to 412–415 nm (ε ranging between 120 mM^−1^ cm^−1^ and 130 mM^−1^ cm^−1^). Such spectroscopic changes reflect the formation of the ferrous nitrosylated *Ma*Pgb* species (*Ma*Pgb*(II)-NO). In fact, the absorption spectrum of the product corresponds to that obtained by adding gaseous NO (∼760 mmHg) to *Ma*Pgb*(II) and mutants in the presence of sodium dithionite (∼1×10^−1^ M).

All *Ma*Pgb*(II)-NO species do not revert to their *Ma*Pgb*(III)-cyanide derivatives; in fact the spectra of all *Ma*Pgb*(II)-NO species reverts to those of the corresponding *Ma*Pgb*(II) derivatives, instead of those of the *Ma*Pgb*(III) forms, by merely pumping off gaseous NO, or bubbling helium through the *Ma*Pgb*(II)-NO solutions. However, the denitrosylation process requires about 12 hours to be completed.

Over the whole NO concentration range explored (1.0×10^−4^ M to 1.0×10^−3^ M), the time course of cyanide dissociation from all *Ma*Pgb*(III)-cyanide species (*i.e.*, for *Ma*Pgb*(III)-cyanide reductive nitrosylation) corresponds to a mono-exponential process for more than 80% of its course (Figure S3A). As reported for horse heart Mb [21], values of the first-order rate constant for reductive nitrosylation of *Ma*Pgb*(III)-cyanide species are independent of the observation wavelength (Figure S3A) and NO concentration. Moreover, the static difference absorption spectra of *Ma*Pgb*(III)-cyanide *minus Ma*Pgb*(II)-NO proteins match very well those obtained kinetically (Figure S3B). Accordingly, the *Ma*Pgb*(III), *Ma*Pgb*(III)-NO, and deoxygenated *Ma*Pgb*(II) species were never detected spectrophotometrically. These findings suggest that cyanide dissociation from all *Ma*Pgb*(III)-cyanide species represents the rate-limiting step of reductive nitrosylation, as described in Scheme 1. Moreover, the first-order rate constant for reductive nitrosylation of all *Ma*Pgb*(III)-cyanide species corresponds to the first-order rate constant for cyanide dissociation, *i.e. k*_off_ [21], [22].

Inspection of Table 1 indicates that the stabilization of the *Ma*Pgb*(III)-cyanide complexes, as monitored by the *k*_off_ values, reflects H-bonding of the ligand to haem distal residue(s), *i.e.* Trp(60)B9 and Tyr(61)B10. In fact, values of *k*_off_ for cyanide dissociation from cyanide-bound *Ma*Pgb*(III) and Leu(142)G4Ala and from the Ile(149)G11Phe ferric mutants, all characterized by ligand stabilization through two H-bonds (to Trp(60)B9 and Tyr(61)B10), are lower than those reported for ligand dissociation from the Tyr(61)B10Ala and Trp(60)B9Ala *Ma*Pgb*(III)-cyanide species, characterized by ligand stabilization through one H-bond. Higher accessibility to the haem-distal ligand-binding site due to the Tyr(61)B10Ala and Trp(60)B9Ala mutations may also partly contribute for the increase in *k_off_* for cyanide.

**Table 1:** Values of the first-order rate constant (*k*_off_) for cyanide dissociation from *Ma*Pgb*(III)- and mutant-cyanide complexes as well as horse heart Mb(III)-cyanide, at pH 9.2 and 20.0°C.

Protein	*k*_off_ (s^−1^)	Number of H-bonds	Residues H-bonded to cyanide
*Ma*Pgb*(III)	(5.8±0.4)×10^−5^	2	Trp(60)B9, Tyr(61)B10
Trp(60)B9Ala	(5.7±0.4)×10^−4^	1	Tyr(61)B10
Tyr(61)B10Ala	(4.2±0.3)×10^−4^	1	Trp(60)B9
Phe(93)E11Leu	(6.3±0.5)×10^−5^	1	Trp(60)B9
Ile(142)G4Ala	(5.1±0.5)×10^−5^	2	Trp(60)B9, Tyr(61)B10
Ile(149)G11Phe	(6.1±0.6)×10^−5^	2	Trp(60)B9, Tyr(61)B10
Horse heart Mb [ref. 21]	(4.9±0.4)×10^−4^	1	His(64)E7

A peculiar case is represented by the Phe(93)E11Leu mutant, where the measured *k*_off_ value for cyanide dissociation suggests that the bound ligand should be stabilized by two H-bonds. However, the crystal structure reveals only the presence of one H-bond, between cyanide and the Nε1 atom of Trp(60)B9 side chain. Analysis of the crystal structure shows that the mutated Leu(93)E11 side chain imposes to the Tyr(61)B10 side chain a slightly unfavorable location for H-bonding to the haem-bound cyanide (the OH group of residue Tyr(61)B10 falls at 4.25 Å on average from the haem-bound cyanide, in the three independent subunits). However, no intervening steric impediments are present for the formation of such H-bond. Indeed, inspection of difference Fourier maps reveals the presence of a positive electron density peak (∼3.8 σ) between the Tyr(61)B10 side chain and the haem-bound cyanide in one of three Phe(93)E11Leu mutant molecules present in the crystal asymmetric unit. Such observation suggests that even in the static crystal environment a fraction of the mutant molecules may stabilize the haem-bound cyanide through two H-bonds. It is therefore reasonable to expect that in a more dynamic environment, as in solution, Tyr(61)B10 side chain could provide the second H-bond to the haem-bound cyanide, in keeping with the observed *k*_off_ value for cyanide dissociation (Table 1).

Remarkably, the values of *k*_off_ for cyanide dissociation from Trp(60)B9Ala *Ma*Pgb*(III)-cyanide and Tyr(61)B10Ala *Ma*Pgb*(III)-cyanide species are closely similar to those reported for cyanide dissociation from Mb(III)-cyanide and Hb(III)-cyanide complexes, where ligand stabilization is achieved through the distal HisE7 residue [21]–[24]. The difference in activation energy for cyanide dissociation from *Ma*Pgb*(III), as well as from ferric Trp(60)B9Ala, Tyr(61)B10Ala, Phe(93)E11Leu, Leu(142)G4Ala, and Ile(149)G11Phe mutants is ∼4 kJ/mol, which is in keeping with the free energy change associated with the formation of ±1 H-bond.

### Azide binding mode to MaPgb*(III)

The size and plasticity of the *Ma*Pgb*(III) distal site [25] suggest that the protein could bind haem ligands bigger than “classical” diatomic species. Therefore, a triatomic molecule such as azide was tested. The structure of the *Ma*Pgb*(III)-azide complex was solved at 1.8 Å resolution (two *Ma*Pgb* molecules in the asymmetric unit) and refined to a final R-factor of 18.9% and R-free of 25.6% (Table S1). The *Ma*Pgb*(III)-azide structure is almost superimposable in its backbone to that of *Ma*Pgb*(III)-cyanide (rms deviation values calculated for 190 Cα atom pairs ranging between 0.26 and 0.39 Å), and the dimeric quaternary structure is preserved.

**Figure 5:**
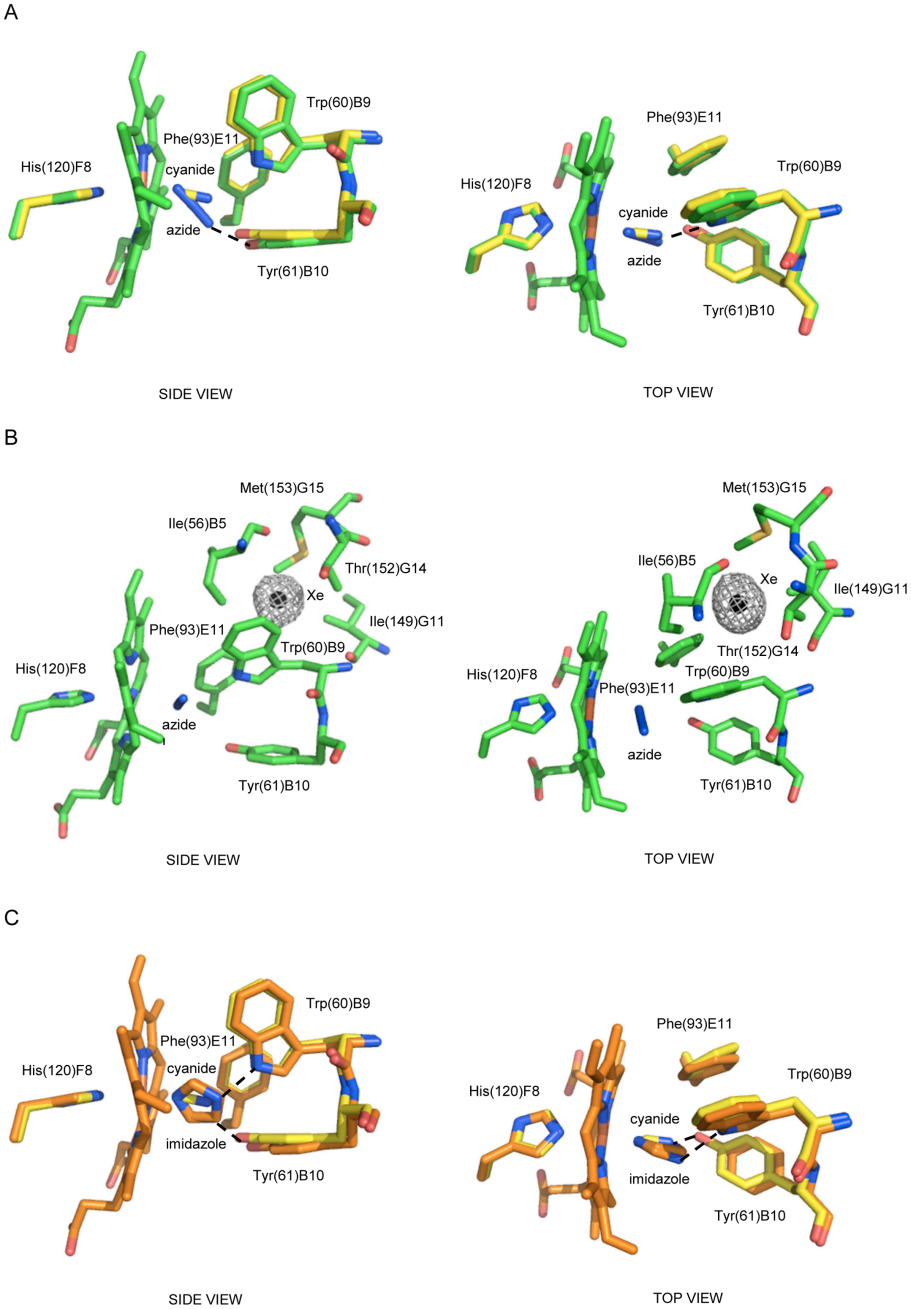
The haem distal site of *Ma*Pgb*(III) in complex with azide, azide and Xenon, and imidazole. Residues lining the haem distal pocket are indicated and shown in stick representation (green) for the *Ma*Pgb*(III)-azide structure, and (orange) for the *Ma*Pgb*(III)-imidazole structure. (**A**) Superimposition of *Ma*Pgb*(III)-cyanide (yellow) onto the *Ma*Pgb*(III)-azide structure. (**B**) Xenon binding inside tunne1. The Xe atom is shown as a black sphere with the corresponding electron density (2Fo-Fc map contoured at 1σ) shown as grey mesh. (**C**) Superimposition of *Ma*Pgb*(III)-cyanide (yellow) onto the *Ma*Pgb*(III)-imidazole structure (the imidazole molecule is shown in two alternate binding modes). In all panels, the proximal His(120)F8 residue is also shown, with the H-bonds to the haem-Fe(III)-bound ligands indicated by dashed lines. All panels are shown from side and top views.

The linear azide anionic ligand is coordinated to the haem-Fe(III) atom with a coordination bond of 1.97 Å for the A chain, and 1.99 Å for the B chain, and is oriented towards tunnel 2, with the Fe–N1–N3 angles of 125° and 114°, respectively. Contrary to the *Ma*Pgb*(III)-cyanide adduct, the haem-Fe(III)-bound azide is stabilized by only one H-bond, provided by the Tyr(61)B10 OH group (2.71 Å for chain A, and 2.72 Å for chain B). The Trp(60)B9 side chain, however, adopts the same orientation in the *Ma*Pgb*(III)-cyanide and in the *Ma*Pgb*(III)-azide complexes. Thus, the absence of a stabilizing H-bond interaction between azide and the Trp(60)B9 side chain can only be ascribed to the triatomic nature of the ligand, which positions its N3 atom ∼4 Å away from the Nε2 atom of Trp(60)B9 (Figure 5A, and Figure S2B). All residues lining the haem distal side match the position found in the *Ma*Pgb*(III)-cyanide structure, comprising the “ligand-sensor” Phe(93)E11. Only Tyr(61)B10 is slightly shifted (about 0.9 Å at the OH group) due to the different size of the bound ligand (azide *vs* cyanide) (Figure 5A). Furthermore, similarly to the *Ma*Pgb*(III)-cyanide structure, the Cα-backbone at the 150–154 region diverges significantly relative to that of the ligand-free *Ma*Pgb*(III) and *Ma*Pgb*(II)-O_2_ structures [6], with structural heterogeneity at the Thr(150)G12 side chain (Figure 2).

### Xenon binding to MaPgb*(III)-azide and -cyanide complexes

To examine the accessibility of the apolar tunnels, a *Ma*Pgb*(III)-azide crystal was equilibrated with high pressures of pure Xe gas, following an approach that has been used successfully to identify cavities in a variety of Mbs and Hbs [26]–[29]. Specifically, the *Ma*Pgb*(III)-azide crystal was exposed to 10 atm of Xenon (for ∼10 min), and a full diffraction data set on the *Ma*Pgb*(III)-azide-Xe derivative was successfully collected at 2.3 Å resolution. The resulting structure was refined to a final R-factor of 22.0% and R-free of 27.4% (Table S1). A similar Xenon-binding experiment was performed also on the *Ma*Pgb*(III)-cyanide derivative, but after treatment under Xenon pressure the crystals diffracted to a resolution lower (3 Å, see Table S1) than the *Ma*Pgb*(III)-azide-Xe crystal, which is therefore reported here to illustrate the Xenon-binding analysis. All results discussed below apply however also to the *Ma*Pgb*(III)-cyanide-Xe derivative structure (Figure S4).

The backbone structure of *Ma*Pgb*(III)-azide-Xe is virtually identical to that of *Ma*Pgb*(III)-azide, with rms deviations ranging from 0.27 Å to 0.28 Å, depending on the superimposed subunits, calculated for all 190 Cα atom pairs. In particular the haem cavities and the two tunnel-systems are identical (Figures 5A and B). The only noticeable difference is the orientation of the azide ligand in the haem distal site pocket, which is found almost parallel to the haem plane (Fe-N1 distance of 2.76 Å and 2.78 Å found in chains A and B of the X-ray structure, respectively), oriented roughly along the line connecting the pyrrole N_A_ and N_C_ nitrogen atoms. The azide N2 atom is weakly H-bonded to the Trp(60)B9 Nε2 atom (3.36 Å for chain A, and 3.22 Å for chain B), and the Tyr(61)B10 OH group falls too far from the ligand to provide any H-bond (∼4 Å from the azide N1 atom) (Figure 5B). Such ligand-haem arrangement indicates the occurrence of a pentacoordinated haem structure. The absence of ligand coordination to the haem-Fe atom may arise from X-ray-induced Fe(III)→Fe(II) reduction, resulting essentially in drastic loss of haem affinity for the ligand, as noticed for several different haem proteins [23]. A similar photoreduction effect is also found in the *Ma*Pgb*(III)-cyanide-Xe complex, where the cyanide ion is at 2.8 Å from the haem-iron atom, and it is stabilized only by a weak hydrogen bond to the Trp(60)B9 NE2 atom (3.38 Å) (Figure S4).

Inspection of the residual difference electron density, after the initial refinement of Xe-bound *Ma*Pgb*(III)-azide, indicates the presence of one Xe atom at refined occupancy of ∼60% and temperature factors of 46.5 Å^2^. The Xe-binding site is located inside tunnel 1, trapped in a hydrophobic pocket resulting from closure of tunnel 1 operated by Trp(60)B9 side chain. The bound Xe atom is stabilized by favorable van der Waals contacts with Ile(56)B5, Trp(60)B9, Phe(93)E11, Ile(149)G11, Thr(152)G14, and Met(153)G15 (Figure 5B and Figure S4). No Xe atoms have been detected in tunnel 2, likely due to its short length and more polar nature.

### Imidazole binding mode to MaPgb*(III)

To verify whether molecules larger than azide could be coordinated to the *Ma*Pgb* haem-Fe(III) atom, imidazole binding was tested on the crystalline protein. The structure of the *Ma*Pgb*(III)-imidazole complex was solved at 1.38 Å resolution (one *Ma*Pgb* molecule in the asymmetric unit) and refined to final R-factor of 15.9% and R-free of 19.8% (Table S1). The structure of *Ma*Pgb*(III)-imidazole is well superimposable in its backbone to that of the *Ma*Pgb*(III)-cyanide complex (rms deviation values calculated for 190 Cα atom pairs ranging between 0.41 Å and 0.45 Å, depending on the superimposed subunits), and their dimeric quaternary structure are identical.

The azimuthal orientation of the haem-Fe(III)-bound imidazole is staggered relative to the haem pyrrole nitrogen atoms (lying at about 15° from a line connecting the methinic CHA-CHC atoms), with a haem-Fe(III) coordination bond of 1.97 Å. The haem-Fe(III)-bound imidazole was refined in a double conformation (occupancy of 0.5 each) with the imidazole N3 atom pointing alternatively towards the Tyr(B10)61 OH group (2.69 Å distance), and the Nε1 atom of Trp(B9)60 (2.64 Å distance) (Figure 5C, and Figure S2C). Additional stabilizing interactions are provided by several van der Waals contacts involving the hydrophobic *Ma*Pgb*(III) haem distal site residues, such as Val(B13)64, Phe(CD1)74, Val(E7)89, Phe(E11)93, and Phe(G7)145, surrounding the haem-Fe(III)-bound imidazole.

Overall, the haem distal site structure of the *Ma*Pgb*(III)-imidazole complex superimposes well with that of *Ma*Pgb*(III)-cyanide. Small differences occur in the position of the Tyr(61)B10 OH group (a shift of 0.75 Å), and the rotation of the Phe(93)E11 side chain around the Cβ-Cγ bond (of ∼13°).

Thus, the ligand sensing mechanism of the Phe(93)E11 is preserved and the Trp(B9)60 side chain is properly oriented in the haem distal site cavity to stabilize the haem-Fe(III)-bound imidazole and to shut tunnel 1. It should be noted, however, that residual positive (Fo – Fc) electron density is present in the region where Trp(60)B9 side chain is located in ligand-free *Ma*Pgb*(III), thus suggesting that in a low percentage of the protein molecules (in the crystal) tunnel 1 could still be open. As found in the *Ma*Pgb*(III)-cyanide and *Ma*Pgb*(III)-azide structures, the 150–154 region, which faces the Trp(60)B9 side chain, is markedly divergent in the Cα-backbone relative to the ligand-free *Ma*Pgb*(III) and *Ma*Pgb*(II)-O_2_ structures, and is affected by structural heterogeneity, with Thr(150)G12 and Met(153)G15 in two alternate conformations (Figure 2).

### Nicotinamide binding mode to MaPgb*(III)

In search for a *Ma*Pgb*(III) ligand larger than imidazole, we tested nicotinamide, based on the knowledge that certain globins, such as leghemoglobins, can bind nicotinic acid [23], [29].

**Figure 6:**
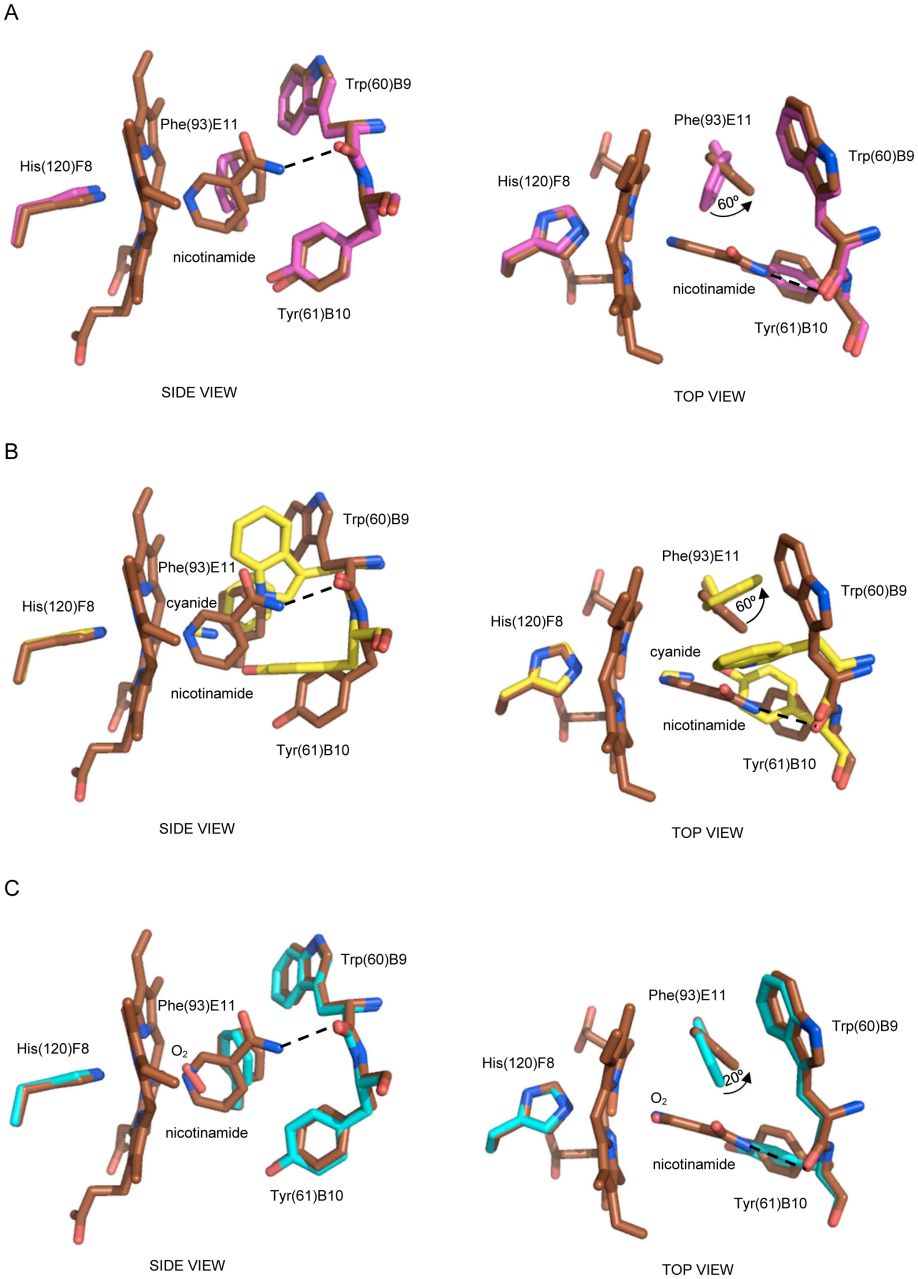
The haem distal site of *Ma*Pgb*(III)-nicotinamide. Residues lining the haem distal pocket are indicated and shown in stick representation (brown). Superimposition of *Ma*Pgb*(III)-nicotinamide onto (**A**) ligand-free *Ma*Pgb*(III) (magenta), (**B**) *Ma*Pgb*(III)-cyanide (yellow), and (**C**) *Ma*Pgb*(II)-O_2_ (cyan) structures. All panels are shown from side and top views. The proximal His(120)F8 residue is also shown. Rotation of the Phe(93)E11 side chain upon ligand binding is indicated in each top view panel. H-bonds to the haem-Fe(III)-bound ligands are shown as dashed lines.

The structure of the *Ma*Pgb*(III)-nicotinamide complex was solved at 1.90 Å (two *Ma*Pgb* molecules in the asymmetric unit) and refined to final R-factor and R-free values of 17.7% and 21.1%, respectively (Table S1). The *Ma*Pgb*(III)-nicotinamide structure appears particularly interesting because although ligand binding neither affects significantly the tertiary structure of the protein (rms deviation values range between 0.39 Å and 0.48 Å, calculated for 190 Cα atom pairs relative to *Ma*Pgb*(III)-cyanide) nor the quaternary dimer assembly, it reshapes the haem distal cavity in a manner which is intermediate between those of the ligand-free *Ma*Pgb*(III) and the *Ma*Pgb*(III)-cyanide, *Ma*Pgb*(III)-azide, and *Ma*Pgb*(III)-imidazole structures (Figure 6A).

In the *Ma*Pgb*(III)-nicotinamide complex (two subunits present in the crystal asymmetric unit) the N atom of the heterocyclic compound is coordinated to the haem-Fe(III) atom at 2.15 Å in chain A, and 2.08 Å in chain B. The amide group points toward the interior of the distal site cavity, in the direction of tunnel 1, with the N atom H-bonded to the carbonyl oxygen of Trp(60)B9. The side chain of Phe(93)E11 provides stacking interaction with the heterocycle of nicotinamide, being rotated of ∼60° relative to the ligand-free *Ma*Pgb*(III) structure (Figure 6A, and Figure S2D). This location is intermediate between those of the ligand-free and cyanide-bound *Ma*Pgb*(III), where cyanide binding induces a rotation of about 120° to the Phe(93)E11 side chain (Figure 6B). Interestingly, the position of the Phe(93)E11 side chain in the *Ma*Pgb*(III)-nicotinamide complex is similar to that found in *Ma*Pgb*(II)-O_2_ [6], with a difference of ∼20° (Figure 6C). As observed in the *Ma*Pgb*(II)-O_2_ structure [6], the rotation of the Phe(93)E11 side chain found in the *Ma*Pgb*(III)-nicotinamide crystalline form is not sufficient to allow the insertion of residue Trp(60)B9 into the haem distal cavity; as a result, tunnel 1 remains open. The Tyr(61)B10 side chain is not involved in ligand stabilization, but H-bonded to Arg(90)E8 peptidic N and to Leu(86)E4 carbonyl O atoms, as observed in the *Ma*Pgb*(II)-O_2_ structure [6]. Thus, although the nicotinamide molecule is the largest ligand relative to cyanide, azide and imidazole, its binding results in a smaller rotation of Phe(93)E11, not sufficient to trigger closure of tunnel 1 by residue Trp(60)B9. Therefore, Phe(93)E11 side chain appears to sense the nature rather than the size of the haem-Fe(III)-bound ligand. It must also be noted that, despite the binding of the bulky nicotinamide ligand, the Cα backbone at the 150–154 region matches well those of the ligand-free *Ma*Pgb*(III) and *Ma*Pgb(II)-O_2_ [6], with no signs of structural heterogeneity (Figure 2).

## Discussion

In order to analyse the ligand binding mechanisms in *Ma*Pgb, we solved the crystal structures of cyanide-, azide-, imidazole-, and nicotinamide-bound *Ma*Pgb*(III). Moreover, the role of selected mutations (Trp(60)B9Ala, Tyr(61)B10Ala, Phe(93)E11Leu, Leu(142)G4Ala, and Ile(149)G11Phe) on cyanide binding to *Ma*Pgb*(III) was examined from both the structural and the kinetic viewpoints. Such structural analyses allowed us to identify new features within the haem distal site, which depict ligand recognition mechanisms that appear unique among those of known globins.

Firstly, in consideration of the strong hydrophobicity of the haem distal site, all ligands are productively stabilized by H-bonds, which are provided by aromatic residue side chains. The prototypical case is represented by the haem-Fe(III)-bound cyanide, which is stabilized by two H-bonds provided by Trp(60)B9 and Tyr(61)B10 side chains. While ligand stabilization by the OH group of Tyr(61)B10 requires only one side chain rotation relative to the ligand-free *Ma*Pgb*(III), the H-bond provided by the Nε2 atom of Trp(60)B9 requires a complex rearrangement of the haem distal site cavity (Figure 1).

Secondly, Phe(93)E11 appears to play the role in ligand sensing and discrimination. In fact, the Phe(93)E11 side chain is able to rotate by ∼120°, relative to ligand-free *Ma*Pgb*(III), upon cyanide binding (Figure 1A, right panel). A similar Phe(93)E11 side chain rotation occurs upon azide and imidazole binding (Figure 5A and C, right panel). Therefore, ligand binding to the ferric form brings about a ligand-linked conformational change which leads first to a rotation of the Phe(93)E11 side chain; this movement, which is observed for cyanide, azide and imidazole binding (but it is somehow impaired in the case of nicotinamide) creates then the space for a consequent “induced-fit” structural change of Trp(60)B9, which brings the Trp(60)B9 Nε2 atom at H-bonding distance from the haem Fe(III)-bound ligand. Such a sequential mechanism is confirmed by the structure of the ferric cyanide-bound Trp(60)B9Ala mutant, where the ligand is coordinated to the haem-Fe(III) atom and stabilized only by the Tyr(61)B10 OH group, the Phe(93)E11 residue being rotated by about 120° relative to ligand-free *Ma*Pgb*(III), even in the absence of Trp(60)B9 (Figure 3A). Modelling considerations suggest that the minimal rotation of the Phe(93)E11 side chain able to trigger the Trp(60)B9 conformational change should be higher than 60°, in agreement with the inspection of the *Ma*Pgb*(III)-nicotinamide complex structure, where, despite the 60° rotation of the Phe(93)E11 side chain, the Trp(60)B9 residue does not enter the haem distal cavity and does not shut tunnel 1 (Figure 6A). Such distal site structural arrangement is reminiscent of that found in the *Ma*Pgb(II)*-O_2_ structure [6], where Trp(60)B9 does not enter the distal site cavity thus leaving tunnel 1 open (Figure 6C). The ligand-sensing role of Phe(93)E11 does not necessarily require the presence of an aromatic residue at the E11 topological position as demonstrated by the structure of the cyanide-bound *Ma*Pgb*(III) Phe(93)E11Leu mutant, where the ligand binding mode and the distal site geometry are essentially identical to those found in the *Ma*Pgb*(III)-cyanide structure (Figure 4A). Furthermore, the presence of the Trp(60)B9 side chain within the haem distal site affects the orientation of the haem-Fe(III)-bound cyanide, but does not impact on the ability of the adjacent Tyr(61)B10 residue to reorient its side chain toward the haem-Fe(III)-bound-ligand, as demonstrated by the structure of the cyanide-bound Trp(60)B9Ala mutant (Figure 3A). Similarly, the Tyr(61)B10Ala mutation does neither impair the Phe(93)E11 ligand-sensing mechanism described above, nor ligand stabilization provided by Trp(60)B9 (Figure 3B).

Cyanide dissociation rate constants, measured in solution for *Ma*Pgb*(III) and its mutants (Table 1), are in good agreement with the ligand-stabilization events and mechanisms described by the crystal structures. The only exception is the Phe(93)E11Leu mutant, where two H-bonding interactions are expected from the dissociation kinetics measurements, while only one, to the Nε2 atom of Trp(60)B9, is found in the crystal structure of the cyanide derivative. However, inspection of the crystal structure of the cyanide-bound Phe(93)E11Leu mutant suggests that Tyr(61)B10 OH group is oriented properly towards the haem-Fe(III)-ligand to allow H-bonding with minimal structural/dynamical arrangement of the haem distal site cavity.

Furthermore, the presence of more than one residue, namely Trp(60)B9 and Tyr(61)B10, involved in H-bond interactions with the haem-bound ligand, and the conformational flexibility of the distal site residues which can modulate the H-bond strength, can explain the observed heterogeneity in resonance Raman stretching bands and in the CO dissociation kinetics (our unpublished results). Overall, the comparison of *Ma*Pgb*(III) in its liganded and unliganded states identifies two distinct haem distal site arrangements: (*i*) a closed distal site conformation, whereby residue Trp(60)B9 points towards the haem-Fe(III)-bound ligand and shuts tunnel 1; and (*ii*) an open distal site conformation, where the Trp(60)B9 side chain points away from the distal pocket, thus keeping tunnel 1 in its open state. In addition, it should be noted that for a quite different ligand, such as nicotinamide, the ligation state of the protein does not alter either the general architecture of the haem distal site or the open/closed state of tunnel 1. Thus, the reactivity of *Ma*Pgb might also be regulated by the nature of the haem ligand.

A third important structural aspect, here highlighted, is the relocation of the Trp(60)B9 side chain into the haem distal cavity upon ligand binding, marking the transition of tunnel 1 from the open to the closed state. The structures of *Ma*Pgb*(III)-azide and -cyanide in complex with Xenon clearly show that the Trp(60)B9 side chain efficiently seals the distal site relative to the tunnel 1 entrance, creating a hydrophobic cavity where the Xenon atom can be trapped (Figure 5B and Figure S4). On the contrary, no Xenon atoms bind at tunnel 2, due to its short length and hydrophilic nature. Thus, the accessibility to the haem cavity, at least through tunnel 1, is linked to and possibly modulated by the ligation state of the protein through a complex mechanism of side chain rearrangements which involve three conserved residues at the key topological positions B9, B10, and E11. In fact, the nature of the ligand seems to drive the distal site architecture and the open/closed tunnel 1 state through conformational relocation of the Phe(93)E11 side chain, as shown by the different positioning of residue Phe(93)E11 in the *Ma*Pgb*(III)-nicotinamide complex relative to the cyanide-, azide-, and imidazole-bound adducts (Figure 6). Thus, Pgbs maintain the ligand stabilization mechanism based on residue B10, as most invertebrate globins [23], but they evolved an E7-independent ligand-to-haem path for additional ligand stabilization/recognition, based on residues at the B9 and E11 topological positions, which appear to behave in a sequential “induced-fit” fashion.

The insertion of Trp(60)B9 into the haem distal cavity upon ligand binding was found to be coupled to a change in the backbone structure of the neighbouring 149–154 region with signs of alternate conformations for residues Ile(149)G11, Thr(150)G12, Thr(152)G14, and Met(153)G15 (Figure 2A). The backbone structures of the 149–154 region in cyanide-, azide-, and imidazole-bound *Ma*Pgb*(III) clearly cluster together, in contrast with those found for ligand-free and nicotinamide-bound *Ma*Pgb*(III) as well as *Ma*Pgb*(II)-O_2_ (Figure 2B). The 149–154 region is located in the second half of the G-helix, at the subunit interface of the *Ma*Pgb* homodimer. The association interface is contributed mostly by residues belonging to the G- and H-helices, which build an intermolecular four-helix bundle. However, within the bundle, tight packing involves specifically the N-terminal half of the G-helices and the C-terminal half of the H-helices, while the remaining interface regions are mostly solvent exposed and marginally involved in direct subunit-subunit interactions (Figure 2B). Thus, the 149–154 region can afford some flexibility to compensate the structural changes transmitted from reshaping of the haem distal site upon ligand binding. On the other hand, the 149–154 region might be able to influence/modulate the architecture and ligand binding properties of the haem distal cavity through association of an (unknown) effector molecule or partner protein. Whether such regulation might occur in an allosteric fashion is a further possibility, although (negative) cooperativity has been reported for O_2_ but not for CO binding [6], [30].

The conformational adaptability shown here by *Ma*Pgb* haem distal site residues, together with the size and hydrophobicity of the haem distal cavity, suggest that physiological ligands other than “classical” diatomic molecules can target the haem-protein. Furthermore, the plasticity of the haem distal site residues, resulting in coupling between ligand sensing and haem distal site accessibility through a double tunnel system, strongly supports the idea of a dual path ligand exchange mechanism (typical of some enzymes), whose functional implications, for a yet undiscovered role in *M. acetivorans* CO metabolism, will be object of future investigations.

## Supporting Information

Figure S1 **The** ***Ma*** Pgb* fold. (**A**) The figure highlights the secondary structure elements (gray; labels A through H). The main protein structural elements that are specific of *Ma*Pgb* (relative to 3-on-3 Hbs) are displayed in orange (Z-helix) and in green (N-terminal, CE, and FG loops). Notice the N-terminal region, the CE and FG loops that bury the haem (red) and prevent access of small ligands to the heme distal cavity, which is connected to the solvent region by tunnel 1 (blue mesh) and tunnel 2 (magenta mesh). (**B**) Close up of the *Ma*Pgb* tunnel system. The program Surfnet [31] was used to explore the protein matrix tunnels with a 1.4 Å radius probe. Residues Trp(60)B9 and Tyr(61)B10 at the entrance of tunnel 1 and tunnel 2, respectively, are shown in stick representation (yellow) and labelled.
(TIF)

Figure S2 **Electron density at the haem distal site of** ***Ma*** Pgb*. Stick representation of the distal site of (**A**) *Ma*Pgb*(III)-cyanide (yellow), (**B**) *Ma*Pgb*(III)-azide (green), (**C**) *Ma*Pgb*(III)-imidazole (orange), and (**D**) *Ma*Pgb*(III)-nicotinamide (brown). The electron density (2F_O_-F_C_ map contoured at 1σ: cyan mesh) is shown around Trp(60)B9 and Tyr(61)B10, the haem, and the haem-bound ligands.
(TIF)

Figure S3 **Reductive nitrosylation of the** ***Ma*** Pgb*(III)-cyanide complex, at pH 9.2 and 20.0°C. (**A**) Time courses of *Ma*Pgb*(III)-cyanide reductive nitrosylation at 410 nm and 425 nm (diamonds and squares, respectively). The analysis of data obtained at 410 nm (diamonds) according to Eqn. (1) allowed the determination of *k*_off_ = (5.9±0.2)×10^−5^ s^−1^. The analysis of data obtained at 425 nm (squares) according to Eqn. (2) allowed the determination of *k*_off_ = (5.8±0.2)×10^−5^ s^−1^. (**B**) Difference static and kinetic absorbance spectra of *Ma*Pgb*(III)-cyanide *minus Ma*Pgb*(II)-NO (dotted line and circles, respectively). The final concentration of *Ma*Pgb*(III) was 2.4×10^−6^ M. The final cyanide concentration was ∼2.0×10^−5^ M. The final NO concentration was between 1.0×10^−4^ M and 1.0×10^−3^ M.
(TIF)

Figure S4 **Xenon-binding site in** ***Ma*** Pgb*(III)-azide and -cyanide complexes. Superimposition of *Ma*Pgb*(III)-cyanide (pink) onto the *Ma*Pgb*(III)-azide structure (green). The bound-Xe atom is shown as a sphere in black (*Ma*Pgb*(III)-azide) and in grey (*Ma*Pgb*(III)-cyanide). Residues lining the haem distal pocket and the Xenon-binding cavity are indicated and shown in stick representation. The proximal His(120)F8 residue is also shown. H-bonds are indicated by dashed lines.
(TIF)

Table S1 **Data collection and refinement statistics for various derivative of ferric** ***Ma*** Pgb*.
(DOC)

Table S2 **Data collection and refinement statistics for cyanide derivative of ferric** ***Ma*** Pgb* mutants.
(DOC)
