# Supplementary figures and images for: Structure and Haem-Distal Site Plasticity in Methanosarcina acetivorans Protoglobin
Source: PLoS One. 2013 Jun 12;8(6):e66144. doi: 10.1371/journal.pone.0066144 (PMC3680402; doi:10.1371/journal.pone.0066144)

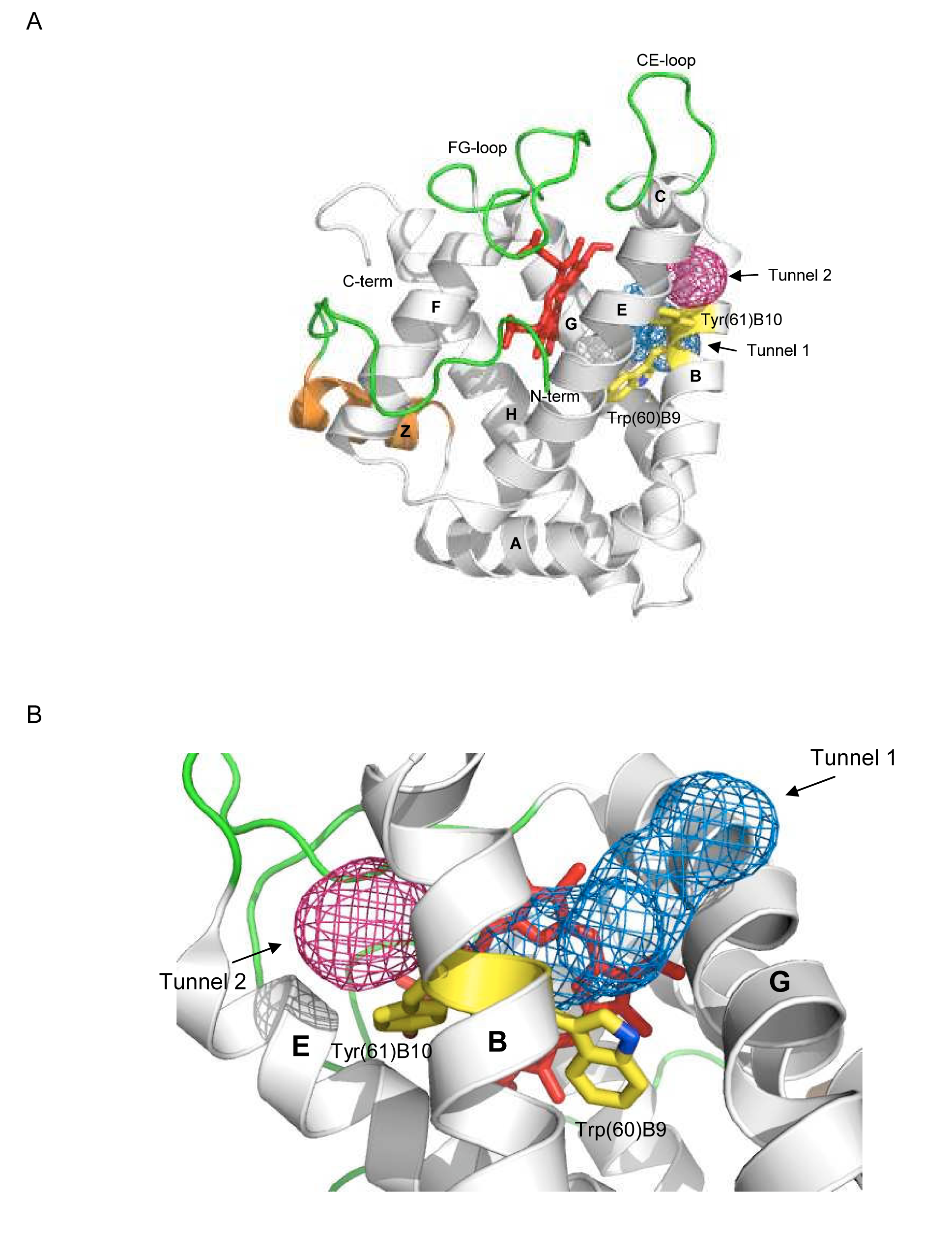

Supplement: Figure S1 — The Ma Pgb* fold. (A) The figure highlights the secondary structure elements (gray; labels A through H). The main protein structural elements that are specific of MaPgb* (relative to 3-on-3 Hbs) are displayed in orange (Z-helix) and in green (N-terminal, CE, and FG loops). Notice the N-terminal region, the CE and FG loops that bury the haem (red) and prevent access of small ligands to the heme distal cavity, which is connected to the solvent region by tunnel 1 (blue mesh) and tunnel 2 (magenta mesh). (B) Close up of the MaPgb* tunnel system. The program Surfnet [31] was used to explore the protein matrix tunnels with a 1.4 Å radius probe. Residues Trp(60)B9 and Tyr(61)B10 at the entrance of tunnel 1 and tunnel 2, respectively, are shown in stick representation (yellow) and labelled. (TIF) [file pone.0066144.s001.tif]

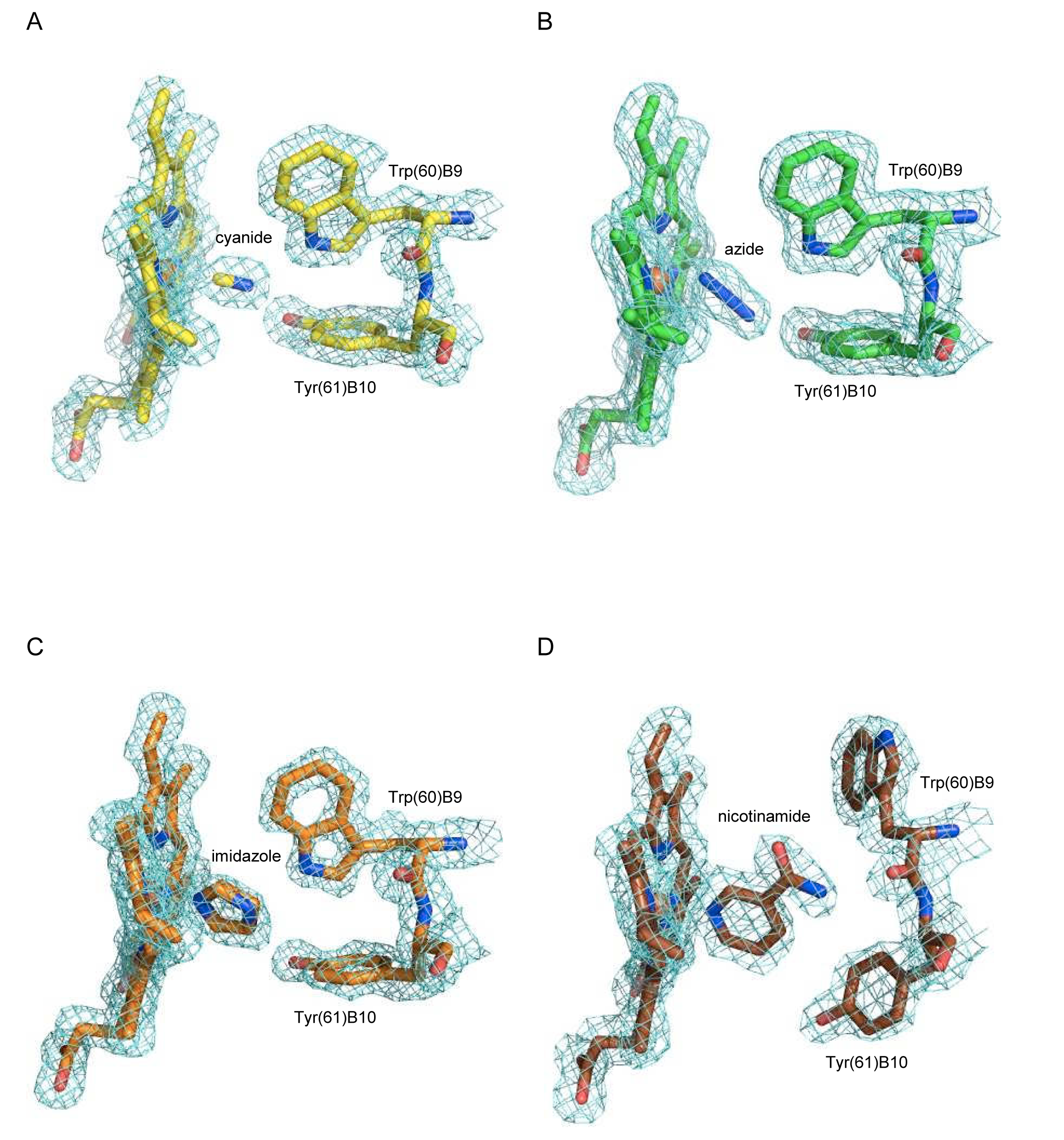

Supplement: Figure S2 — Electron density at the haem distal site of Ma Pgb*. Stick representation of the distal site of (A) MaPgb*(III)-cyanide (yellow), (B) MaPgb*(III)-azide (green), (C) MaPgb*(III)-imidazole (orange), and (D) MaPgb*(III)-nicotinamide (brown). The electron density (2FO-FC map contoured at 1σ: cyan mesh) is shown around Trp(60)B9 and Tyr(61)B10, the haem, and the haem-bound ligands. (TIF) [file pone.0066144.s002.tif]

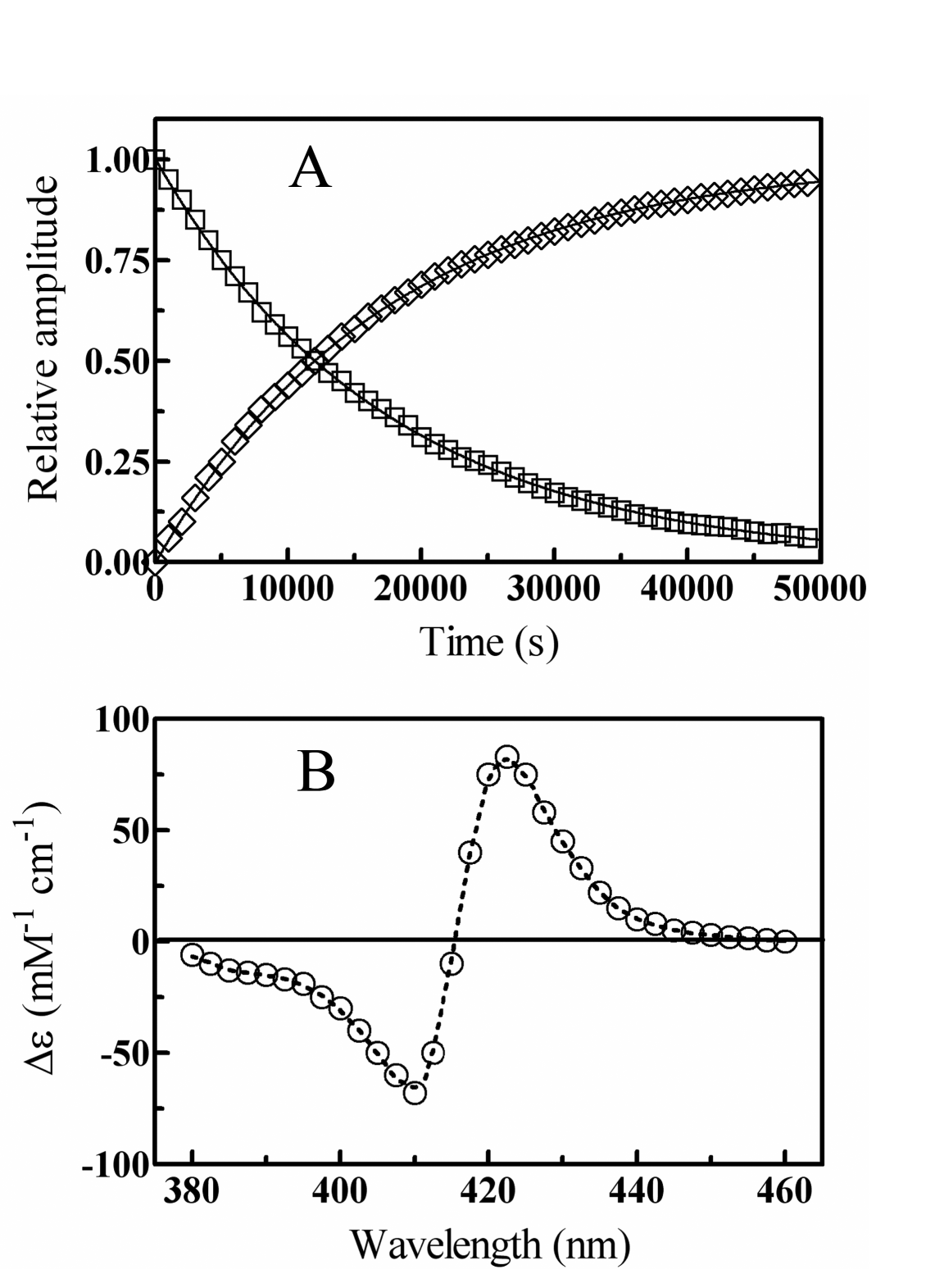

Supplement: Figure S3 — Reductive nitrosylation of the Ma Pgb*(III)-cyanide complex, at pH 9.2 and 20.0°C. (A) Time courses of MaPgb*(III)-cyanide reductive nitrosylation at 410 nm and 425 nm (diamonds and squares, respectively). The analysis of data obtained at 410 nm (diamonds) according to Eqn. (1) allowed the determination of k off = (5.9±0.2)×10−5 s−1. The analysis of data obtained at 425 nm (squares) according to Eqn. (2) allowed the determination of k off = (5.8±0.2)×10−5 s−1. (B) Difference static and kinetic absorbance spectra of MaPgb*(III)-cyanide minus MaPgb*(II)-NO (dotted line and circles, respectively). The final concentration of MaPgb*(III) was 2.4×10^−6^ M. The final cyanide concentration was ∼2.0×10^−5^ M. The final NO concentration was between 1.0×10^−4^ M and 1.0×10^−3^ M. (TIF) [file pone.0066144.s003.tif]

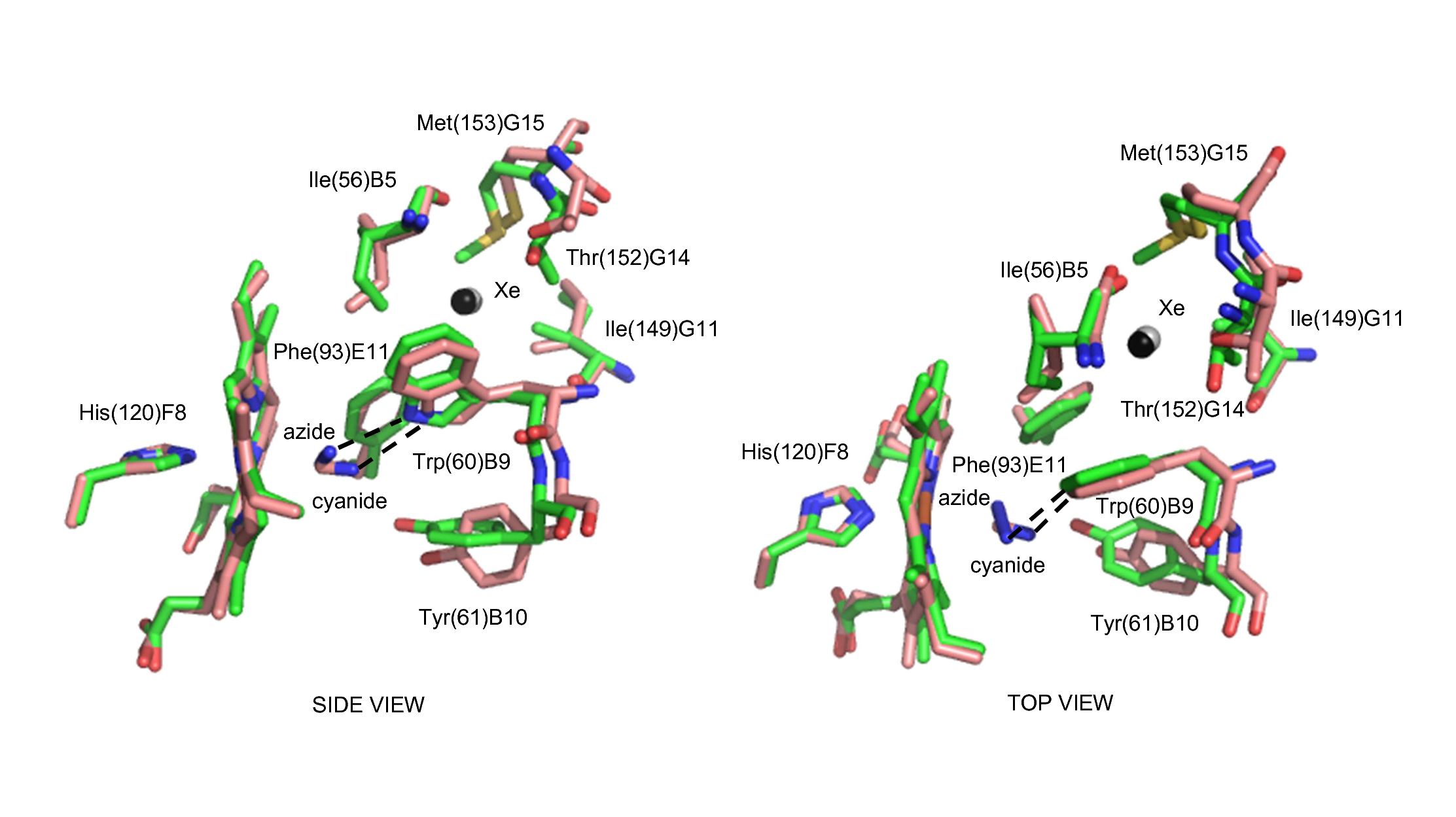

Supplement: Figure S4 — Xenon-binding site in Ma Pgb*(III)-azide and -cyanide complexes. Superimposition of MaPgb*(III)-cyanide (pink) onto the MaPgb*(III)-azide structure (green). The bound-Xe atom is shown as a sphere in black (MaPgb*(III)-azide) and in grey (MaPgb*(III)-cyanide). Residues lining the haem distal pocket and the Xenon-binding cavity are indicated and shown in stick representation. The proximal His(120)F8 residue is also shown. H-bonds are indicated by dashed lines. (TIF) [file pone.0066144.s004.tif]
