# Supplementary material for: Structure and Haem-Distal Site Plasticity in Methanosarcina acetivorans Protoglobin
Source: PLoS One. 2013 Jun 12;8(6):e66144. doi: 10.1371/journal.pone.0066144 (PMC3680402; doi:10.1371/journal.pone.0066144)
Supplement: Table S1 — Data collection and refinement statistics for various derivative of ferric Ma Pgb*. (DOC) [file pone.0066144.s005.doc]

**Supplementary Table S1.** Data collection and refinement statistics for various derivative of ferric *Ma*Pgb*.

|  | ***Ma*Pgb*-cyanide** | ***Ma*Pgb*-azide** | ***Ma*Pgb*-imidazole** | ***Ma*Pgb-nicotinamide** | ***Ma*Pgb*-cyanide-Xenon** | ***Ma*Pgb*-azide-Xenon** |
| --- | --- | --- | --- | --- | --- | --- |
| ***Data collection*** |  |  |  |  |  |  |
| Synchrotron beamline | ESRF ID14-1 | ESRF ID14-4 | ESRF ID14-1 | ESRF ID23-1 | ESRF ID14-1 | ESRF ID14-4 |
| Temperature | 100 K | 100 K | 100 K | 100 K | 100 K | 100 K |
| Space group | *P*2_1_ | *C*2 | *C*2 | *P*2_1_ | *C*2 | *P*2_1_ |
| Cell dimensions | *a* = 49.15 Å, | *a* = 119.88 Å, | *a* = 79.97 Å, | *a* = 50.53 Å, | *a* = 80.66 Å, | *a* = 50.98 Å, |
|  | *b =* 47.96 Å, | *b =* 48.82 Å, | *b =* 49.06 Å, | *b =* 48.21 Å, | *b =* 50.16 Å, | *b =* 48.12 Å, |
|  | *c* = 80.29 Å, | *c* = 80.59 Å, | *c* = 51.43 Å, | *c* = 81.01 Å, | *c* = 51.45 Å, | *c* = 80.49 Å, |
|  | *β* = 106.7° | *β* = 122.6° | *β* = 92.5° | *β* = 102.8° | *β* = 100.6° | *β* = 94.6° |
| Resolution (Å) | 26.32-1.60 | 41.62-1.80 | 23.41-1.38 | 49.28-1.90 | 50.64-3.00 | 41.45-2.30 |
|  | (1.69-1.60)*^a^* | (1.90-1.80) | (1.45-1.38) | (2.00-1.90) | (3.16-3.00) | (2.42-2.30) |
| Observations | 184,540 | 122,823 | 95,313 | 117,068 | 6,963 | 40,811 |
| Unique reflections | 44,547 | 35,389 | 40,771 | 30,234 | 3,627 | 17,255 |
| Completeness (%) | 94.2 (93.1) | 97.1 (97.5) | 98.9 (97.8) | 99.9 (100.0) | 87.8 (91.3) | 98.8 (99.6) |
| R-merge*^b^*(%) | 10.1 (16.1) | 7.5 (39.7) | 5.2 (25.7) | 10.0 (31.1) | 13.6 (29.7) | 6.9 (12.8) |
| I/σ(I) | 10.4 (7.0) | 8.4 (2.8) | 13.1 (3.4) | 10.6 (4.8) | 6.4 (2.3) | 6.6 (4.8) |
| Multiplicity | 4.1 (4.1) | 3.5 (3.5) | 2.3 (2.3) | 3.9 (3.9) | 1.9 (1.9) | 2.4 (2.4) |
| ***Refinement*** |  |  |  |  |  |  |
| R-factor/R-free (%) | 17.5/23.8 | 18.9/25.6 | 15.9/19.8 | 17.7/21.1 | 20.4/30.6 | 22.0/27.4 |
| Protein residues in the a.u. | 190 (A)*^c^*, 191 (B) | 190 (A), 190 (B) | 191 (A) | 190 (A), 191 (B) | 190 (A) | 191 (A), 191 (B) |
| Heme groups | 2 | 2 | 1 | 2 | 1 | 2 |
| Water molecules | 439 | 298 | 252 | 311 | 22 | 105 |
| Fe^3+^-bound ligand | 2 | 2 | 1 | 2 | 1 | 2 |
| Xenon | - | - | - | - | 1 | 2 |
| Glycerol | 5 | - | 6 | 1 | - | - |
| Phosphate ions | 1 | - | 2 | - | - | - |
| ***Model quality*** |  |  |  |  |  |  |
| Overall B-factor (Å^2^) | 9.0 (A), 9.7 (B) | 23.2 (A), 28.8 (B) | 12.0 (A) | 12.7 (A), 11.4 (B) | 54.1 (A) | 21.7 (A), 21.7 (B) |
| Rmsd from ideal values: |  |  |  |  |  |  |
| bond lengths (Å) | 0.017 | 0.015 | 0.010 | 0.008 | 0.011 | 0.011 |
| bond angles (°) | 1.6 | 1.4 | 1.3 | 1.1 | 2.2 | 1.2 |
| Ramachandran plot: |  |  |  |  |  |  |
| most favored regions | 94.1 | 93.2 | 91.7 | 94.1 | 86.3 | 92.0 |
| additional allowed regions | 5.9 | 6.8 | 8.3 | 5.9 | 13.7 | 8.0 |

*^a^* Outer shell statistics are shown within parentheses

*^b^* R-merge =Σ_h_Σ_i_ | I_hi_ – <I_h_> | / Σ_h_Σ_i_ I_hi_

*^c^* *Ma*Pgb* subunit in the crystal asymmetric unit
