# Supplementary material for: Structure and Haem-Distal Site Plasticity in Methanosarcina acetivorans Protoglobin
Source: PLoS One. 2013 Jun 12;8(6):e66144. doi: 10.1371/journal.pone.0066144 (PMC3680402; doi:10.1371/journal.pone.0066144)
Supplement: Table S2 — Data collection and refinement statistics for cyanide derivative of ferric Ma Pgb* mutants. (DOC) [file pone.0066144.s006.doc]

**Supplementary Table S2.** Data collection and refinement statistics for cyanide derivative of ferric *Ma*Pgb* mutants.

|  | **Trp(60)B9Ala** | **Tyr(61)B10Ala** | **Phe(93)E11Leu** | **Leu(142)G4Ala** | **Ile(149)G11Phe** |
| --- | --- | --- | --- | --- | --- |
| ***Data collection*** |  |  |  |  |  |
| Synchrotron beamline | ESRF ID23-1 | ESRF ID14-1 | ESRF ID14-1 | ESRF ID14-1 | ESRF ID14-1 |
| Temperature | 100 K | 100 K | 100 K | 100 K | 100 K |
| Space group | *P*2_1_ | *P*2_1_ | *C*2 | *P*2_1_ | *C*2 |
| Cell dimensions | a = 48.51 Å, | a = 50.45 Å, | a = 124.90 Å, | a = 50.61 Å, | a = 124.70 Å, |
|  | b = 47.66 Å, | b = 47.96 Å, | b = 48.29 Å, | b = 48.07 Å, | b = 48.03 Å, |
|  | c = 80.22 Å, | c = 80.42 Å, | c = 99.12 Å, | c = 80.35 Å, | c = 98.68 Å, |
|  | *β* = 107.2° | *β* = 102.0° | *β* = 94.6° | *β* = 101.9° | *β* = 94.2° |
| Resolution (Å) | 29.86-1.70 | 26.22-1.50 | 37.26-2.00 | 26.21-1.50 | 26.14-1.50 |
|  | (1.79-1.70)*^a^* | (1.58-1.50) | (2.11-2.00) | (1.58-1.50) | (1.58-1.50) |
| Observations | 154,391 | 212,648 | 120,162 | 192,162 | 302,559 |
| Unique reflections | 38,690 | 57,199 | 39,922 | 58,766 | 92,938 |
| Completeness (%) | 99.8 (100.0) | 94.7 (96.8) | 99.4 (99.6) | 96.9 (99.6) | 99.5 (99.7) |
| R-merge*^b^*(%) | 11.3 (41.3) | 5.7 (8.5) | 14.2 (38.4) | 4.0 (8.2) | 6.8 (38.0) |
| I/σ(I) | 8.7 (3.1) | 16.4 (11.1) | 5.6 (2.8) | 23.5 (13.0) | 10.0 (3.0) |
| Multiplicity | 4.0 (4.0) | 3.7 (3.8) | 3.0 (3.0) | 3.3 (3.3) | 3.3 (3.2) |
| ***Refinement*** |  |  |  |  |  |
| R-factor/R-free (%) | 14.9/21.5 | 13.5/16.9 | 20.2/26.2 | 12.0/16.4 | 17.7/22.2 |
| Protein residues in the a.u. | 191 (A)*^c^*, 193 (B) | 192 (A), 191 (B) | 190 (A), 190 (B), 190 (C) | 191 (A), 191 (B) | 191 (A), 191 (B), 191 (C) |
| Heme groups | 2 | 2 | 3 | 2 | 3 |
| Water molecules | 311 | 406 | 362 | 491 | 477 |
| Cyanide ion | 2 | 2 | 3 | 2 | 3 |
| Glycerol | - | 3 | 7 | - | 3 |
| Isopropanol | - | - | 2 | - | 1 |
| Hepes | - | - | 1 | - | 2 |
| ***Model quality*** |  |  |  |  |  |
| Overall B-factor (Å^2^) | 12.0 (A), 12.0 (B) | 8.7 (A), 9.4 (B) | 21.5 (A), 18.3 (B), 18.2 (C) | 9.8 (A), 10.3 (B) | 19.2 (A), 14.9 (B), 18.1 (C) |
| Rmsd from ideal values: |  |  |  |  |  |
| bond lengths (Å) | 0.010 | 0.009 | 0.015 | 0.012 | 0.016 |
| bond angles (°) | 1.2 | 1.3 | 1.5 | 1.3 | 1.6 |
| Ramachandran plot: |  |  |  |  |  |
| most favored regions | 94.4 | 94.4 | 93.5 | 94.7 | 94.1 |
| additional allowed regions | 5.6 | 5.6 | 6.5 | 5.3 | 5.9 |

*^a^* Outer shell statistics are shown within parentheses

*^b^* R-merge =Σ_h_Σ_i_ | I_hi_ – <I_h_> | / Σ_h_Σ_i_ I_hi_

*^c^* *Ma*Pgb* subunit in the crystal asymmetric unit
